# Differentiation of soil metabolic function and microbial communities between plantations and natural reforestation

**DOI:** 10.3389/fmicb.2025.1544641

**Published:** 2025-02-28

**Authors:** Nannan Zhang, Xiaoxia Chen, Tingju Ren, Jiangcheng Luo, Jin Liang, En Tao Wang, Fusun Shi

**Affiliations:** ^1^Chengdu Institute of Biology, Chinese Academy of Sciences, Chengdu, China; ^2^CAS Key Laboratory of Mountain Ecological Restoration and Bioresource Utilization and Ecological Restoration and Biodiversity Conservation Key Laboratory of Sichuan Province, Chengdu Institute of Biology, Chinese Academy of Sciences, Chengdu, China; ^3^University of Chinese Academy of Sciences, Beijing, China; ^4^College of Life Science, Sichuan Normal University, Chengdu, China; ^5^Escuela Nacional de Ciencias Biológicas, Instituto Politécnico Nacional, Ciudad de México, Mexico

**Keywords:** microbiome, metabolomics, plant-microbial interaction, mono-planation, natural reforestation

## Abstract

Reforestation plays a vital role in restoring the soil degradation areas. However, the mechanisms by which different restoration approaches affect the soil properties and microbial communities remain unclear. Aiming to understand the interactions between plant species, soil properties, and microbial communities in different restoration approaches, we investigated the soil microbial community using nontargeted metabolomics to explore how the reforestation approach affects soil physicochemical properties, soil metabolites, and soil microbial communities. The results showed that the reforestation approach, soil layer, and their interactive effects significantly affected soil organic carbon, total nitrogen, dissolved organic carbon, available phosphorus concentrations, and root traits. The diversity and composition of bacterial and fungal communities in natural reforestation (NR) were different from those in artificial mono-plantations, and their network interactions were more significant in NR than in artificial plantations. A clear separation of metabolites between the artificial plantations and NR was observed in the soil metabolite analysis. Two pathways, linoleic acid metabolism, and valine, leucine, and isoleucine biosynthesis, were significantly regulated between the artificial mono-plantations and NR. Different soil traits were significantly correlated with dominant microbial taxa in the four reforestation approaches. 13-L-hydroperoxylinoleic acid, 13-S-hydroxyoctadecadienoic acid, homovanillin, and 9,10-epoxyoctadecenoic acid showed the highest correlation with the microbial taxa in the network. Partial least squares path modeling (PLS-PM) shows that root-mediated soil physicochemical properties were the primary factors affecting the bacterial community among the reforestation approaches. The soil fungal community is directly regulated by plant roots in the subsoil and indirectly regulated by the root-mediated physicochemical properties in the topsoil. We conclude that different reforestation approaches affect the soil microbial community through root and soil physicochemical properties rather than soil metabolites.

## 1 Introduction

Reforestation effectively controls soil degradation, improves soil fertility, and restores the ecosystem services and functions of degraded forest lands (Veldkamp et al., 2020; Sakti et al., 2024; Zhang et al., 2024). Restored plantations cover more than 200 million ha worldwide, of which 25% are comprised of fast-growing tree species (FRA, 2010). The effects of reforestation on soil functions and properties vary among plant species (Chen et al., 2019; Brown et al., 2023). The composition of tree species and stand structure in artificial forests were lower than those in natural secondary forests. The high growth rates of artificial reforests can result in increased demand for soil nutrients, a slower rate of litter decomposition, and an imbalance between nutrient inputs and outputs (Liu W. et al., 2023; Kaźmierczak et al., 2024), ultimately leading to soil degradation (Liu et al., 2024). This difference may be related to plant traits that alter soil properties and microbial communities, which are critical mediators of many processes linked to plant health, soil productivity, and multiple ecosystem functions (Wille et al., 2019; Mueller et al., 2020; Shi et al., 2024). Thus, understanding how different plants mediate plant–soil–microbe interactions can provide insight into the different feedback mechanisms of reforestation.

The composition and diversity of soil microbes are influenced by biotic (i.e., soil fauna activity, root exudates, microbial interactions, plant communities) and abiotic (such as temperature, soil physicochemical properties, and geographic and climatic conditions) factors (Beugnon et al., 2023; Chamard et al., 2024). Soil physicochemical properties such as pH and organic matter can influence the structure of the soil microbiome (Philippot et al., 2023; Zhang et al., 2023), whereas plants can regulate microbial activity and community composition in the rhizosphere through the secretion of root exudates or bioactive molecules (Dhungana et al., 2023). Compared with adjacent secondary forests, artificial forests can alter the availability of soil nutrients due to changes in the quantity and quality of plant material entering the soil via root exudates and leaf litter, which always cause alterations in soil microbial communities and thus may further affect plant–soil–microbe interactions (Chen et al., 2019; Spitzer et al., 2021). For example, Chen et al. (2019) compared the composition of nitrogen-fixing microorganisms in soils planted with legumes and non-legumes and found that the abundance of diazotrophs in the soil with legume (*Acacia mangium*) plantations was greater than that in the soil of non-legume (*Pinus massoniana*) plantations.

Soil metabolites from soil organic matter, plant tissues, soil animals, and microorganisms play crucial roles in regulating many processes, such as microbial activities (Cheng et al., 2022). These metabolites serve as sources of energy and nutrients for microorganisms and regulate microbial growth, function, and diversity (Bi et al., 2021; Hao et al., 2022). Bi et al. (2021) reported that plants release certain fatty acids and secondary metabolites (such as diterpenoids) to regulate bacterial and fungal community composition. Variations in the chemical phenotypes of roots among tree species can modify the responses exhibited by rhizosphere microbes, ultimately demonstrating a conditioning effect of plants on soil chemical composition (Mueller et al., 2020). Soil metabolomics, an emerging and powerful technology, provides insights into the coupling of organic/inorganic compounds and soil microbial communities (Song et al., 2020). However, regarding the reforestation approach, the mechanisms of how different restoration approaches affect the microbial community, particularly the relationships among the root traits, soil physicochemical properties, soil metabolites, and soil microbial community structure in different forest restoration approaches, have yet to be thoroughly explored. Therefore, combining the analysis of microbial community composition with the composition and functional metabolic pathways of soil metabolites could provide a more comprehensive understanding of intricate biological processes in soil (Morton et al., 2019). This integration of knowledge is crucial for informed decision-making in the ecological restoration of damaged ecosystems.

Therefore, the main objectives of the present study were: (a) to clarify the effects of different reforestation approaches on root traits, soil physicochemical properties, soil metabolites, and microbial communities, to examine the interaction patterns of plant–soil microbes using a reforestation approach; and (b) to determine the relative importance of soil physicochemical properties and metabolites in shaping the microbial community under different reforestation approaches. This study provides new insights into the systematic coupling of soil metabolomics and microbial communities during forest reforestation.

## 2 Materials and methods

### 2.1 Sampling site and sampling

This study was carried out at the Maoxian Mountain Ecosystem Research Station (103°54′ E, 31°42′ N), Chinese Academy of Sciences, in Sichuan Province, located on the eastern edge of the Tibetan Plateau, which has an annual precipitation of 850 mm and a mean annual temperature of 9.3°C, with Calcic Luvisol soil type according to the IUSS Working Group (WRB, 2006). The natural forest in the area was felled on a large scale from the 1940s to the 1980s (Wang et al., 2016). Reforestation was conducted to develop plantations in the 1980s, with approximately 60% of the land in the area. Four reforestation approaches were applied to the degraded region. One approach was natural reforestation (NR) and the other three were artificial plantations of evergreen coniferous tree spruce (*Picea asperata* Mast.) with a shallow root system, pine (*Pinus tabuliformis Carrière*) with a deep root system, and broad-leaved deciduous tree katsura (*Cecidiphyllum japonicum* Sieb. and Zucc.), which is an important ecological and economic tree species designed for formonoculture plantations (Hu et al., 2016). The dominant species of naturally regenerating forests are the broad-leaved deciduous trees *Quercus aliena* Blume and *Corylopsis willmottiae* Rehd. and Wils, *Berberis candidula* Jytte and the bamboo *Fargesia nitida* (Mitford ex Stapf) Keng f. ex T. P. Yi (GRIN). The forest sites have not received any management since planting, although they are frequently disturbed by the extraction of Chinese medicinal plants and wild mushrooms in the spring.

In August 2022, three field plots (20 m × 20 m) were selected within each reforestation approach, and each field plot was approximately 40 m from the adjacent plot. Field sampling was conducted in three 1 m × 1 m subplots, randomly selected from each plot. To avoid the disturbance of dead roots, the subplots were at least 0.8 m from any stumps. Each subplot sample was collected with five soil cores (20 cm in depth, 7 cm in diameter) from depths of 0–20 cm (topsoil) and 40–60 cm (subsoil) and mixed into one composite sample. Roots in each sample were separated from the soil by sieving through a 5-mm mesh and stored at 4°C after washing with tap water.

In total, 24 soil samples (3 replicates × 4 four reforestation approaches × 2 soil layers) were obtained. Each soil sample was sieved (<2 mm) and divided into three portions: one portion was stored at −20°C for DNA extraction, one was stored at 4°C for analysis of microbial biomass, and the other was air-dried for soil physicochemical analysis.

### 2.2 Analysis of soil physicochemical properties

Soil pH was determined in a 1:2.5 soil:water suspension. The air-dried soil samples (passed through a 0.15-mm sieve) were used for chemical characterization and soil organic carbon (SOC) and total nitrogen (TN) contents were assayed using a vario MACRO cube CN Elemental analyzer (Elementar Analysensysteme, Langenselbold Germany) (Wang et al., 2016). The concentrations of dissolved organic carbon (DOC) and dissolved organic nitrogen (DON) in the extracted solution were determined using a total organic carbon (TOC) analyzer (Shimadzu TOC-VCSH/TN, Kyoto, Japan). Available phosphorus (AP) in the soil was estimated using the Olsen method (Olsen, 1954) and was determined by Inductively Coupled Plasma–Optical Emission Spectroscopy (ICP-OES). Soil microbial biomass carbon (MBC) and microbial biomass nitrogen (MBN) were determined using the fumigation extraction method (Vance et al., 1987) and calculated using the formula: MBC (or MBN) = Fc (or Fn)/kc (or Kn), where Fc (or Fn) = [C (or N) evolved from fumigated soil - C (or N) evolved from non-fumigated soil]; and kc = 0.45 (kn = 0.54) that is the proportion of obtained microbial C (or N) in the total biomass C (or N). The units of MBC, Fc, CO_2_-C and MBN were all in μg C or N g^−1^ soil.

### 2.3 Root morphology measurements

Roots were placed in a thin layer of water on a glass container without overlapping and scanned at a resolution of 600 dpi using a flatbed scanner (Epson Expression 1200XL). Following scanning, the roots were oven-dried at 65°C for at least 48 h and weighed to obtain their dry mass. Scanned images of the roots were analyzed using the WinRHIZO professional root analysis system (Pro 2016a version, Regen Instruments, Canada), which was used to measure root length, surface area, average diameter, and volume. Four root traits were used for analysis: average diameter (AD), specific root length (SRL) = root length/dry weight (cm/g), specific root surface area (SRA) = surface area/dry weight (cm^2^/g), and root tissue density (RTD) = dry weight/volume (g/cm^3^).

### 2.4 Microbial community analyses

Soil DNA was extracted using an EZNATM Omega Mag-bind Soil DNA kit (OMEGA, USA). DNA concentration and quality were evaluated using a NanoDrop Spectrophotometer (Thermo Fisher Scientific, USA) and electrophoresis on 1% (w/v) agarose gel. Primers 338F-806R (Cui et al., 2017) for bacteria and ITS1F-ITS2 (Jiang et al., 2017) for fungi were used for the amplification. Specific methods for PCR amplification and final sequencing are described in detail in Supplementary material (Text 1). All raw sequences were deposited at the National Microbiology Data Center (NMDC) with accession numbers NMDC 40055004–40,055,027 for bacteria and NMDC40055028-NMDC 40055051 for fungi.

### 2.5 Untargeted metabolomic analysis

0.2 g soil were extracted with 600 μL MeOH containing 4 mg/mL 2-amino-3-(2-chloro-phenyl)-propionic acid (Vasilev et al., 2016). The mixture was then placed in a tissue grinder for 90 s at 60 Hz and sonicated for 30 min at room temperature and 30 min on ice. After that, the mixture was centrifuged at 12,000 rpm for 10 min at 4°C, and the supernatant was filtered through a 0.22-μm membrane. The untargeted metabolome was analyzed by Shanghai Personal Biotechnology Co., Ltd. (Shanghai, China) using UHPLC-Q-Exactive MS/MS analysis. Liquid chromatography was performed using a Vanquish UHPLC System (Thermo Fisher Scientific, USA). Chromatographic separation was carried out using an ACQUITY UPLC ® HSS T3 (150 × 2.1 mm, 1.8 μm) (Waters, Milford, MA, USA), maintained at a temperature of 40°C. The mobile phases consisted of (A) 0.1% formic acid in water (A) and 0.1% formic acid in acetonitrile (B). The flow rate was set to 0.25 mL/min. The elution gradient was as follows: 0–1 min, 2% B2; 1–9 min, 2–50% B2; 9–12 min, 50–98% B2; 12–13.5 min, 98% B2; 13.5–14 min, 98–2% B2; 14–20 min, 2% B2 (Zelena et al., 2009). Q Exactive mass spectrometer (Thermo Fisher Scientific, USA) was used for mass spectrometric detection of metabolites.

### 2.6 Statistical analyses

SPSS version 24 was used to calculate descriptive statistical parameters. Soil physicochemical properties, root traits, microbial diversity index (Shannon and Chao1), and taxonomic composition were compared by one-way analysis of variance (ANOVA) with Duncan’s multiple range test. The relative importance of reforestation and soil layer in explaining the variation in environmental variables and microbial community was evaluated using two-way ANOVA. The microbial structure was visualized using principal coordinate analysis (PCoA) of Bray–Curtis distances. Linear discriminant analysis (LDA) effect size (LefSe) was conducted to identify the biomarkers from the phylum to genus level between any artificial plantations and NR and the filter value of the LDA Score to 3.5 (Segata et al., 2011). The effects of soil physicochemical properties and root traits on the soil microbial community were tested using redundancy analysis (RDA) and the function “*envfit*” in the vegan package. PERMANOVA was conducted to test the differences in beta diversity among the four reforestation approaches using the “adonis” function in “vegan” (Anderson, 2006). Differentially expressed metabolites (DEMs) between any artificial plantation and NR were defined as those with variable importance in projection (VIP) > 1.0, *p*-value <0.05, in orthogonal partial least squares analysis (OPLS-DA). The expression of the top 20 DEMs between any artificial plantation and NR is shown using a heatmap. A metabolic pathway with an impact value higher than 0.06 and a *p*-value less than 0.05 is characterized as significantly relevant. Spearman’s correlation coefficients were applied to reveal the correlations between microbial community composition (phylum level), soil properties, and root traits in the R vegan package. The co-occurrence network was employed to explore the associations between soil microbial community structure and soil metabolite profiles. Spearman’s rank correlation coefficients (rs) in the co-occurrence network were considered statistically significant if |r| > 0.7 and *p* < 0.05, as these criteria indicate a strong statistical correlation. To identify the key drivers controlling soil microbial communities, a partial least squares path model (PLS-PM) was used to further reveal the direct and indirect effects of plant roots, soil physicochemical properties, and soil metabolites on soil microbial community (Bi et al., 2021). PLS-PM was performed by using “innerplot” function of the “plspm” package in R (Sanchez, 2013).

## 3 Results

### 3.1 Soil physicochemical properties and root traits

**Figure 1:**
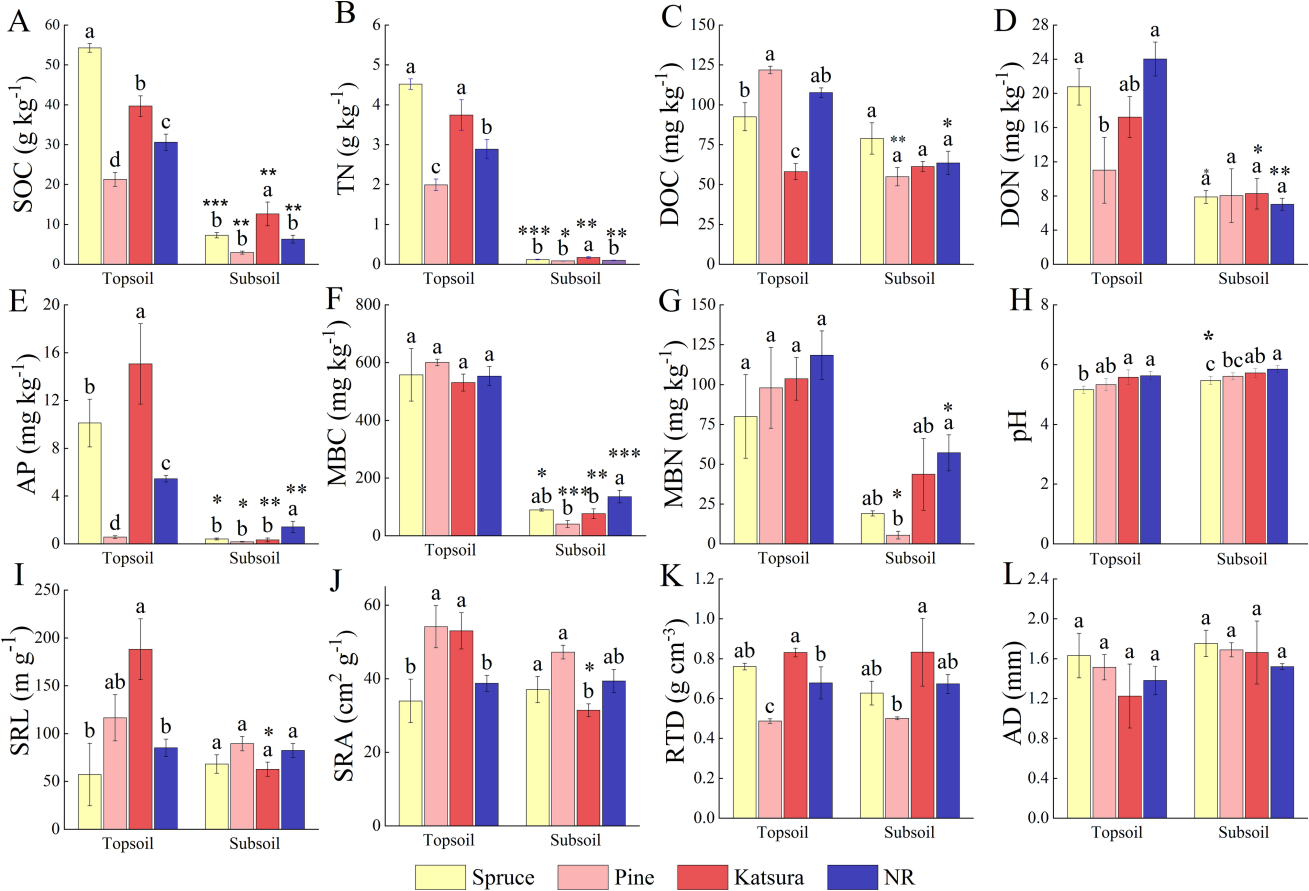
Comparative analysis of soil physicochemical property and root traits in the reforestation approaches corresponding to Spruce, Pine, Katsura, and NR. **(A)** SOC, soil organic carbon; **(B)** TN, total nitrogen; **(C)** DOC, dissolved organic carbon; **(D)** DON, dissolved organic nitrogen; **(E)** AP, available phosphorus; **(F)** MBC, microbial biomass carbon; **(G)** MBN, microbial biomass nitrogen; **(H)** pH; **(I)** SRL, specific root length; **(J)** SRA, specific root surface area; **(K)** RTD, root tissue density; **(L)** AD, average diameter. Values followed by the same letter are not significantly different between reforestation approaches at *p* < 0.05. Asterisk represents significant difference between soil layers within the same reforestation approach (*p* < 0.05). Values are means ± S.E.

According to the association analysis (Table 1), the soil layer significantly affected all the tested soil physicochemical properties; for example, all the nutrient values, except pH, were lower in the subsoil than in the topsoil. The reforestation approach and its interaction with the soil layer exerted significant effects (*p* < 0.001) on SOC, TN, DOC, and AP concentrations. The data in Table 1 also revealed that root traits (SRL and SRA) were significantly influenced by both reforestation and soil layers. The comparative analysis (Figure 1) demonstrated that the contents of SOC, TN, and AP in the topsoil showed the same tendency of spruce > katsura > NR > pine forest, while the contents of DOC, DON, and root traits SRL, SRA, and RTD varied according to the plantation approach. In detail, the contents of DOC in katsura and DON in pine were significantly lower (*p* < 0.05) than those in NR topsoil, while pine forests had significantly lower MBC and MBN contents than NR in the subsoil. Soil pH was lower in spruce than in NR in both the topsoil and subsoil. Katsura forest had significantly higher SRL, SRA, and RTD than NR in the topsoil (Figure 1). There were no significant differences in root characteristics among the different reforestation approaches in the subsoil. Soil SOC, TN, AP, and MBC decreased with increasing soil depth at all reforestations. SRL and SRA values of katsura decreased with increasing soil depth.

**Table 1:** Association probability (*P*) values among the reforestation approach and soil layer and soil properties, root trait and microbial characteristics.

	Reforestation approach		Soil layer		Reforestation approach * Soil layer	
	*F*	*P*	*F*	*P*	*F*	*P*
Soil characteristics						
SOC	43.022	**<0.001**	536.522	**<0.001**	24.299	**<0.001**
TN	24.245	**<0.001**	234.747	**<0.001**	10.360	**<0.001**
DOC	9.371	**0.001**	47.372	**<0.001**	12.568	**<0.001**
DON	2.509	0.096	40.549	**<0.001**	3.309	**0.047**
AP	29.085	**<0.001**	158.757	**<0.001**	30.325	**<0.001**
MBC	0.392	0.760	317.882	**<0.001**	1.293	0.311
MBN	2.244	0.123	31.520	**<0.001**	0.424	0.739
pH	8.635	**0.001**	13.519	**<0.001**	0.360	0.783
Root trait						
SRL	5.447	**0.009**	8.886	**0.009**	7.033	**0.003**
SRA	5.335	**0.010**	4.862	**0.042**	3.920	**0.028**
RTD	7.275	**0.003**	0.360	0.557	0.444	0.725
AD	0.754	0.536	2.436	0.138	0.278	0.840
Bacterial characteristics						
Chao1	8.621	**0.001**	24.176	**<0.001**	1.134	0.365
Shannon	6.119	**0.006**	25.093	**<0.001**	0.302	0.823
Proteobacteria	6.791	**0.004**	28.620	**<0.001**	16.496	**<0.001**
Actinobacteria	8.747	**0.001**	23.370	**<0.001**	0.690	0.571
Acidobacteria	9.154	**0.001**	0.461	0.507	1.009	0.415
Chloroflexi	8.918	**0.001**	40.069	**<0.001**	13.064	**<0.001**
Verrucomicrobia	3.430	**0.042**	13.064	**<0.001**	13.064	**<0.001**
Rokubacteria	0.556	0.651	84.590	**<0.001**	0.876	0.474
Gemmatimonadetes	11.262	**<0.001**	0.599	0.450	3.201	0.052
Firmicutes	6.414	**0.005**	12.106	**0.003**	3.144	0.054
Planctomycetes	0.144	0.932	1.253	0.279	1.804	0.187
Bacteroidetes	6.354	**0.005**	13.671	**0.002**	3.844	**0.030**
Fungal characteristics						
Chao1	35.480	**<0.001**	73.192	**<0.001**	0.813	0.505
Shannon	18.467	**<0.001**	14.426	**0.002**	6.781	**0.004**
Basidiomycota	21.978	**<0.001**	4.502	**0.050**	21.090	**<0.001**
Ascomycota	15.689	**<0.001**	16.928	**0.001**	16.299	**<0.001**
Mortierellomycota	5.440	**0.009**	13.825	**0.002**	2.479	0.098

*p values ≤0.05 are highlighted in bold.*

### 3.2 Composition of the bacterial and fungal community

16S rRNA and ITS gene sequencing yielded 1,372,987 and 1,866,942 effective reads after quality control, ranging from 45,626 to 110,178 reads per sample. As shown in Table 1, association analysis showed that species richness (Chao1 values) and diversity (Shannon index) of both bacterial and fungal communities were significantly associated with reforestation approaches and soil layers. However, the interactive effect of the reforestation approach and soil layer only significantly affected the fungal diversity (Table 1). In a comparison of the topsoils from the tested reforestation approaches (Table 2), NR and katsura forests presented similar Chao1 and Shannon indices of bacteria that were significantly greater (*p* < 0.05) than those in pine and spruce reforestation, whereas the latter two shared similar values. For fungi in topsoils, the Chao1 and Shannon indices showed the following trend: katsura > NR > spruce > pine and katsura > NR > pine > spruce (*p* < 0.05) (Table 2). In the subsoils, the Chao1 index for bacteria presented a similar tendency to that in topsoils: similar values in katsura and NR, which were significantly greater than those of spruce and pine reforestations, but similar Shannon indices were detected among all four reforestation approaches. For fungi, the Chao1 index was the highest in katsura forestry, which differed significantly from that in NR. In comparison between the two soil layers, the Chao1 and Shannon indices of bacteria in subsoils were significantly lower than that in topsoil for the four reforestation approaches, except for the Shannon value in NR. Fungal Chao1 and Shannon indices in katsura and NR and fungal Chao1 in pine forests were also significantly lower in the subsoil than in the topsoil.

**Table 2:** Shannon diversity, Chao1 richness, and coverage of bacterial and fungal communities.

Layers	Treatments	Bacteria			Fungi		
		Chao1	Shannon	Coverage (%)	Chao1	Shannon	Coverage (%)
Topsoil	Spruce	4072.76 ± 230.97b	10.66 ± 0.11b	99.06 ± 0.18	375.13 ± 12.01b	3.56 ± 0.25d	99.97 ± 0.01
	Pine	3903.34 ± 268.13b	10.46 ± 0.12b	99.04 ± 0.13	263.29 ± 9.78c	4.21 ± 0.22c	99.99 ± 0.00
	Katsura	5067.91 ± 280.14a	11.12 ± 0.15a	98.50 ± 0.16	608.98 ± 36.24a	6.44 ± 0.17a	99.97 ± 0.01
	NR	5007.3295 ± 62.92a	11.15 ± 0.17a	98.59 ± 0.07	400.51 ± 16.33b	5.63 ± 0.04b	99.99 ± 0.00
Subsoil	Spruce	3267.44 ± 39.18ab*	9.6 ± 0.11a*	99.57 ± 0.27	238.33 ± 56.94b	4.39 ± 0.35ab	99.99 ± 0.00
	Pine	1812.38 ± 654.52b*	9.45 ± 0.5a*	99.08 ± 0.05	57.54 ± 13.05c*	3.16 ± 0.63b	99.99 ± 0.00
	Katsura	4032.38 ± 176.4a*	10.47 ± 0.11a*	98.7 ± 0.06	380.5 ± 44.1a*	5.05 ± 0.14a*	99.99 ± 0.00
	NR	3762.23 ± 663.19a	10.37 ± 0.41a	99.01 ± 0.29	180.65 ± 37bc*	3.97 ± 0.24ab*	100.00 ± 0.00

Values followed by the same letter are not significantly different between reforestation approaches at *p* < 0.05. ^*^Asterisk represents values significantly lower than the corresponding values in topsoil in the same reforestation approach (*p* < 0.05).

**Figure 2:**
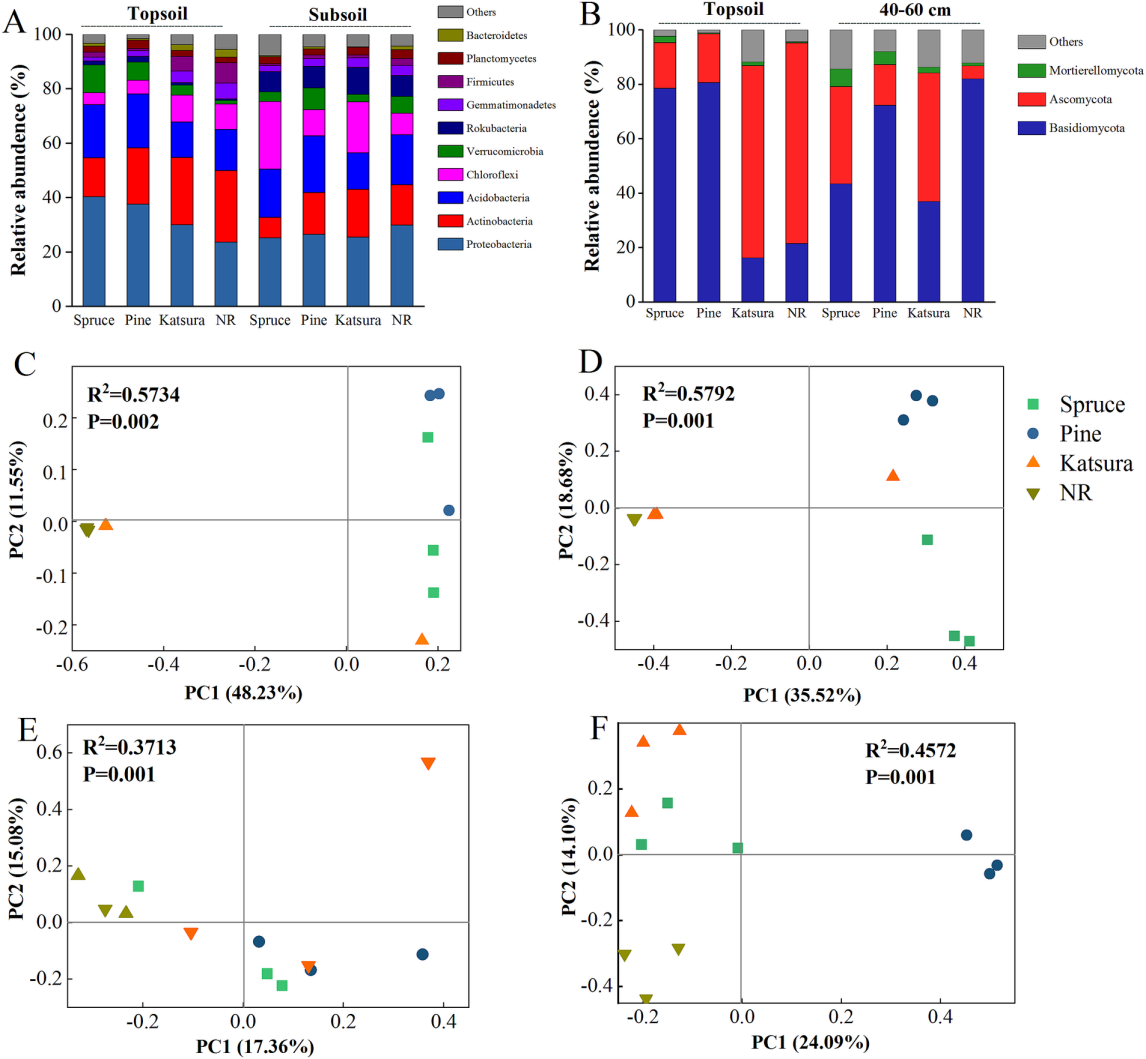
Relative abundances of the dominant bacterial **(A)** and fungal **(B)** phyla in soils of different reforestation approaches. Principal coordinate analysis (PCoA) illustrating changes in bacterial **(C,D)** and fungal **(E,F)** community structure in topsoil and subsoil.

In total, 44 and 11 phyla were identified in the bacterial and fungal sequences, respectively. The dominant bacterial phyla across all soil samples were Proteobacteria (29.78%), Actinobacteria (17.74%), Acidobacteria (17.19%), Chloroflexi (11.25%), and Verrucomicrobia (5.26%), which accounted for more than 81% of all sequences (Figure 2). The dominant fungal phyla were Basidiomycota (53.96%) and Ascomycota (35.27%). The relative abundance of Proteobacteria in the spruce and pine topsoils was enhanced by 70.37 and 58.87%, respectively, whereas the relative abundance of Actinobacteria, Chloroflexi, Gemmatimonadetes, and Bacteroidetes was significantly reduced compared with NR and katsura. The relative abundance of Basidiomycota was 365.17 and 374.90% higher in spruce and pine topsoil, respectively, while the relative abundance of Ascomycota was significantly reduced compared to NR. The relative abundance of Ascomycota and Mucoromycota was 3.09 and 7.39 times higher in spruce and pine subsoils than that in NR. The relative abundances of Proteobacteria, Chloroflexi, Verrucomicrobia, Bacteroidetes, Basidiomycota and Ascomycota were altered by the reforestation approach, soil layer, and the interactive effect of the reforestation approach and soil layer (Table 1). PCoA results indicated that the top two main axes of the bacterial and fungal community structure explained 32.44–59.78% of the variation (Figures 2C–F). The compositions of the bacterial and fungal communities of NR were clearly separated from those of the artificial plantations in the topsoil and subsoil, and the fungal communities of the artificial plantations were distinct from each other. PERMANOVA further revealed that reforestation approaches significantly affected the bacterial and fungal community structures, regardless of the soil layer collected. Bacterial and fungal structures were affected by the soil layer (Supplementary Figure S1).

**Figure 3:**
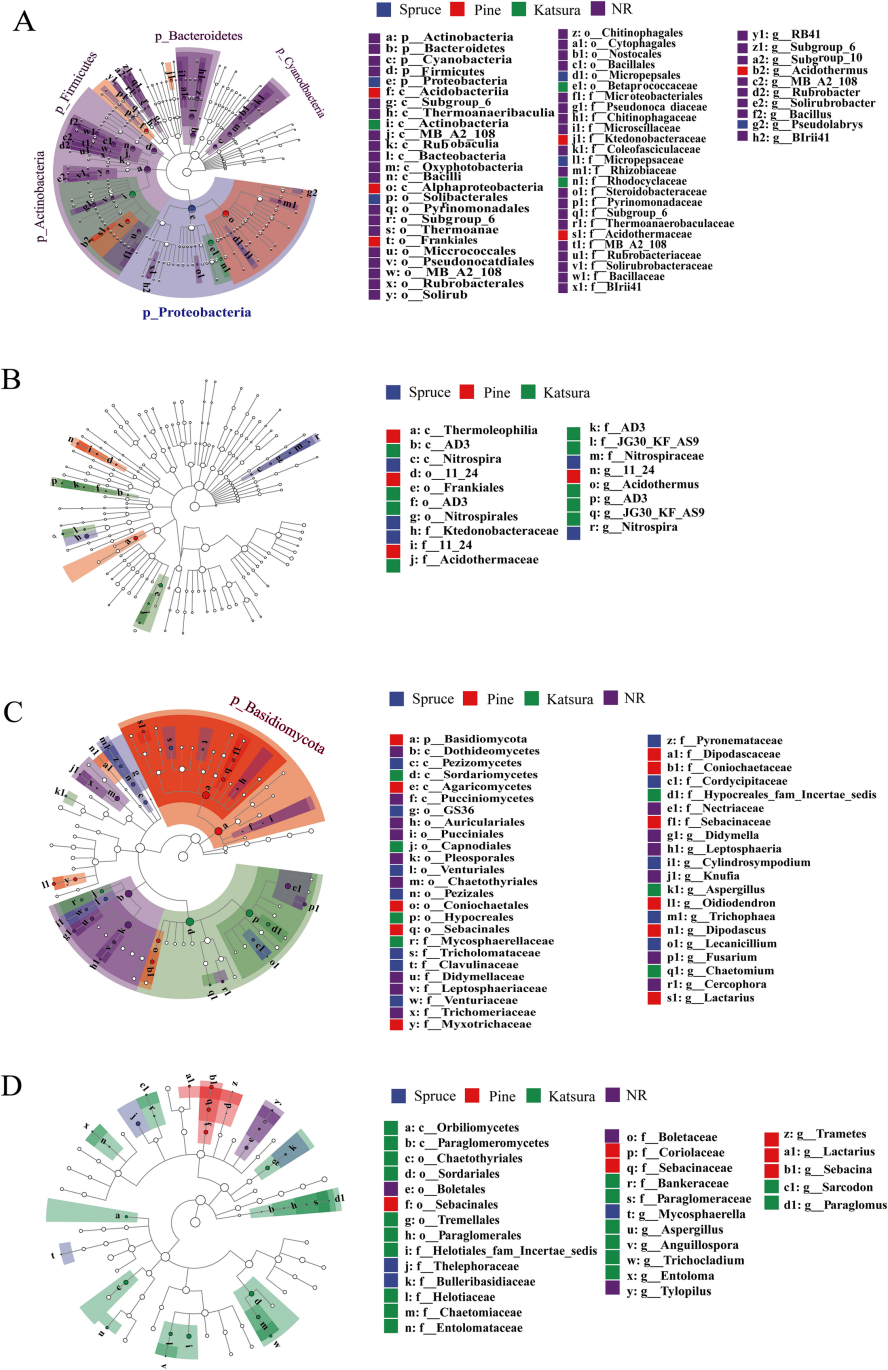
LefSe analysis of soil bacterial **(A,C)** and fungal **(B,D)** community structure in topsoil and subsoil between different reforestation approaches.

**Figure 4:**
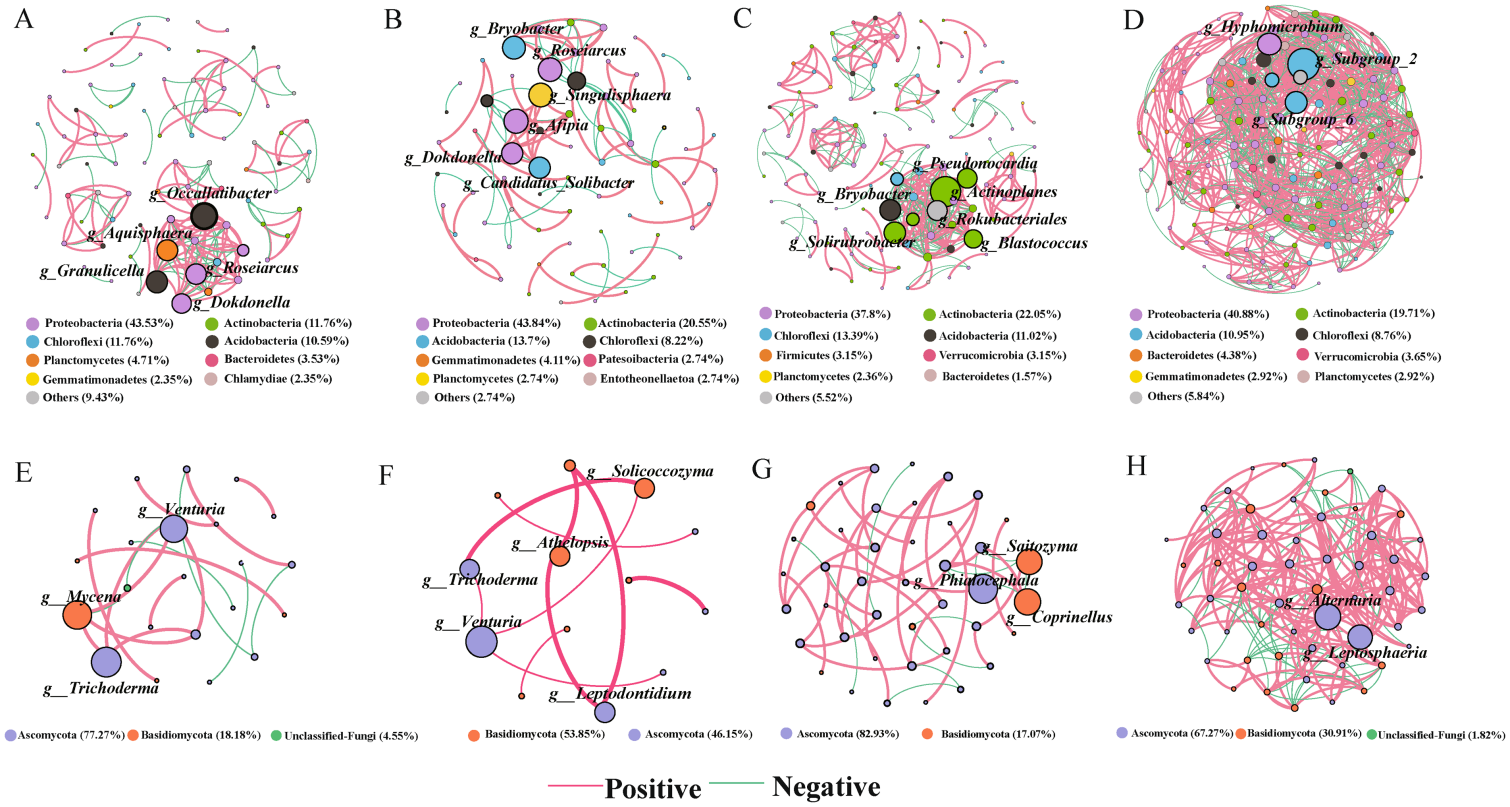
A co-occurrence network of the abundant bacterial taxa **(A–D)** and fungal taxa **(E–H)** in soils of spruce, pine, katsura, and NR, respectively. The node size represented the degree in the network. Only significant Spearman′s correlation coefficients (|r| > 0.7 and *p* < 0.05) are shown. The red lines indicate positive correlations and the green lines indicate negative correlations. The nodes were colored by phylum.

LefSe analysis was used to determine the taxa that could best characterize each biological class. As shown on Figure 3, 60 and 18 bacterial taxa and 47 and 30 fungal taxa were significantly different between the topsoil and subsoil, respectively. There were 45 biomarkers in NR, 6 biomarkers in spruce, 6 biomarkers in pine and 3 biomarkers in katsura in the topsoil. At the phylum level, NR topsoil microorganisms were mainly enriched in Actinobacteria and Firmicutes, including Bacilli (class), Solirubrobacterales (order), and Pseudonocardiales (order). Proteobacteria (phylum), Alphaproteobacteria (class), and Actinobacteria (class) were the most significantly enriched taxa in the spruce, pine, and katsura topsoil, respectively (Supplementary Figure S2A). In the subsoil, the identified biomarkers included Ktedonobacteraceae and *Nitrospira* for spruce, Thermoleophilia for pine, and JG30_KF_AS9 for kasura in the artificial plantations compared to NR (Supplementary Figure S2C). Among the fungi, spruce and pine topsoils significantly increased the abundance of the Tricholomataceae (family) and Basidiomycota (phylum). The most prominent indicators were Sordariomycetes (class) and Pleosporales (order) in katsura and NR topsoils, respectively (Supplementary Figure S2B). NR subsoil was significantly enriched with Boletaceae (family), which differed from that of the artificial plantations. Soil bacterial and fungal networks were generated in the reforestation soils (Figure 4).

Topological properties showed distinguishing differences in taxa correlations between the artificial plantations and NR (Supplementary Table S1). The degree and modularity based on 16S rRNA and ITS genes in katsura soils were higher than those in spruce and pine soils. Additionally, significant differences in keystone taxa (genera) were observed between artificial plantations and NR in the comparison group for the bacterial–fungal networks (Figure 4). The structural properties of the network were greater in NR than in the artificial plantations in both the topsoil and subsoil, indicating more connections and closer relationships of bacterial and fungal taxa under NR. The structural properties of the network were greater in the topsoils than in the subsoils for all four reforestation approaches, indicating more connections and closer relationships of bacterial and fungal taxa in the topsoils.

### 3.3 Soil metabolite characteristics

**Figure 5:**
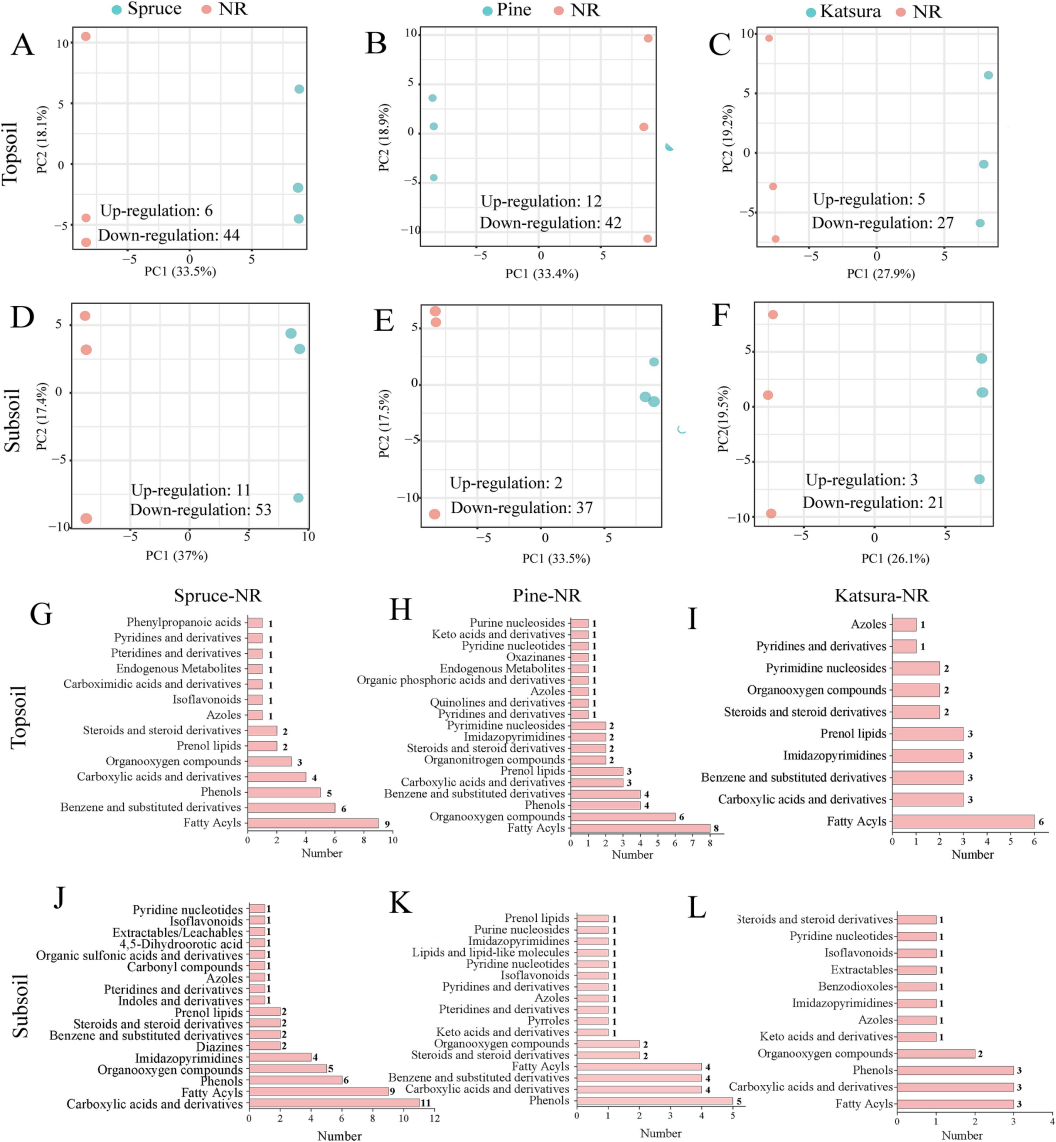
OPLS-DA analysis of metabolites between artificial plantations and NR in topsoil **(A–C)** and subsoil **(D–F)**. Differential metabolite classification statistics between artificial plantations and NR in topsoil **(G–I)** and subsoil **(J–L)**.

In total, 263 metabolites were detected in the samples from the four reforestation approaches by nontargeted metabolomic analysis. The PCoA results (Supplementary Figure S3) revealed no significant difference in the soil metabolite profiles among the four reforestation approaches in both topsoils and subsoils. PERMANOVA further revealed that the soil metabolism spectrum was not considerably altered in response to reforestation (Supplementary Figure S3). The OPLS-DA models were effective and could be used to screen for differentially expressed metabolites (DEMs) (Supplementary Figure S4), which differed between the artificial plantations and the NR. The number of DEMs between each artificial plantation approach and NR presented in the volcano plots (Supplementary Figure S5) clearly showed that the downregulated DEMs were much more abundant than the upregulated ones for all three artificial plantations in both soil layers (detailed data in Figure 5).

PLS-DA results showed clear metabolite separation between the artificial plantations and NR (Figures 5A–F). Compared with that in the corresponding NR samples, 50 and 64 DEMs (6 and 11 upregulated, 44 and 53 downregulated) in the artificial planted spruce forest, 54 and 39 DEMs (12 and 2 upregulated, 42 and 37 downregulated) in the pine forest, and 32 and 24 DEMs (5 and 3 upregulated, 27 and 21 downregulated) in the katsura forest were identified in the topsoil and subsoil, respectively, using the OPLS-DA model (Figures 5A–F). The most enriched DEMs were fatty acyls in the topsoil of the artificial plantations in comparison with NR group (Figures 5G–I). In the subsoil, carboxylic acids and derivatives, phenols, and fatty acyls were the main DEMs for all the three artificial plantations compared with the NR ecosystem (Figures 5J–L). Among the top 20 DEMs in spruce, pine, and katsura forests 2–5 upregulated DEMs in the topsoil and subsoil were presented (Supplementary Figure S6). According to the threshold conditions of impact values, more than 0.06 and *p*-value less than 0.05, differential metabolic pathways were identified for the artificial plantations versus NR. Artificial plantations mainly influenced the metabolite contents in linoleic acid metabolism and valine, leucine, and isoleucine biosynthesis, compared with NR (Supplementary Figure S7). Tyrosine metabolism was only identified in the DEMs of spruce-NR, whereas glycine, serine, and threonine metabolism were only detected in the pine-NR and katsura-NR comparison group.

### 3.4 Relationships between soil physicochemical properties-metabolites and microbial communities

**Figure 6:**
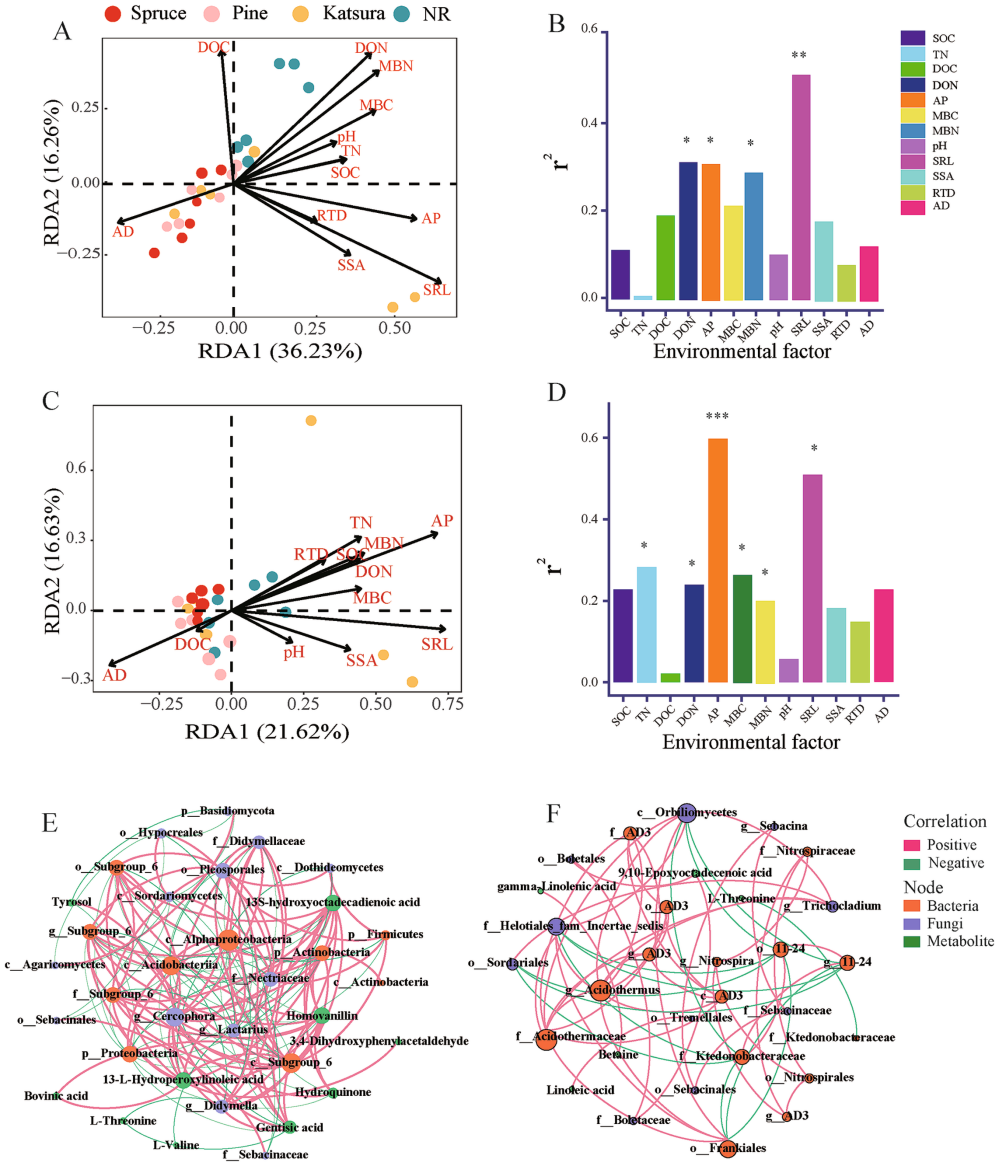
Redundancy analysis (RDA) and envift analysis identified the effects of soil physicochemical property and root traits on bacterial **(A,B)** and fungal **(C,D)** community structures. Co-occurrence network of the differential metabolites and differential microbial taxa in topsoil **(E)** and subsoil **(F)**. The red lines indicate positive correlations and the green lines indicate negative correlations: the thicker the line, the stronger the correlation.

Correlation tests associated the main bacterial and fungal phyla with the soil physicochemical properties and root traits (Supplementary Figure S8) showed that SOC, TN, and MBC concentrations were significantly correlated with Proteobacteria, Chloroflexi, and Rokubacteria; SAL was significantly related to Actinobacteria and Bacteroidetes; DON, AP, and MBN concentrations and RTD were significantly correlated with the dominant fungal phyla Ascomycota. From the Bray–Curtis distances, RDA was used to identify the effects of soil physicochemical properties on bacterial and fungal community structures (Figure 6). RDA showed that SRL, DON, AP, and MBN significantly affected bacterial community composition (Envift analysis; *p* < 0.05), in which AP was the strongest determinant of fungal community composition. AP, SRL, TN, DON, MBC, and MBN were significantly correlated (Envift analysis; *p* < 0.05) with changes in fungal community structures (Figures 6C,D).

Co-occurrence networks were built between the significantly impacted microbial taxa and DEMs (Figures 6E,F). A greater density was found between the microbial taxa and metabolites in the topsoil than in the subsoil. Fungi predominantly connected the network in the topsoil, whereas bacteria were predominant in the subsoil. Alphaproteobacteria and *Cercophora* were the core bacterial and fungal responders, and 13-L-hydroperoxylinoleic acid, 13-S-hydroxyoctadecadienoic acid, and homovanillin (downregulated in artificial plantation - NR comparison group) were prominent metabolite responders that negatively co-occurred with Alphaproteobacteria and positively co-occurred with *Cercophora* in topsoil (Figure 6E; Supplementary Table S2). *Acidothermus* and Orbiliomycetes were the core bacterial and fungal responders in subsoil. 9,10-epoxyoctadecenoic acid in the subsoil of artificial plantation NR was the prominent metabolite responder that positively co-occurred with fungi in the order Boletales.

**Figure 7:**
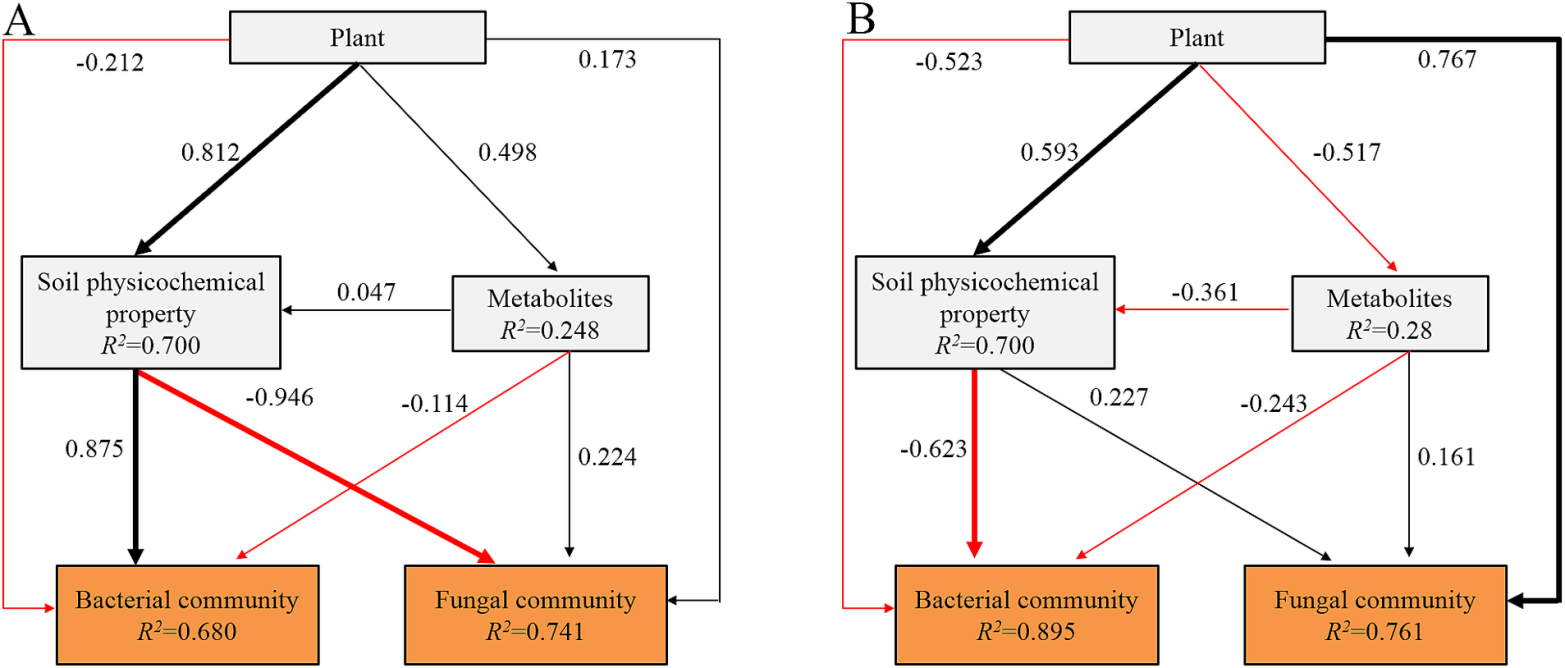
The partial least squares path model (PLS-PM) showing the direct and indirect effects of plants, soil physicochemical property, and soil metabolites on bacterial and fungal communities in topsoil **(A)** and subsoil **(B)**. The blue and red arrows indicate the positive and negative flow of causality. Bold arrows indicate significant standardized paths (*p* < 0.05). The number on the arrow indicates the effective normalized path coefficient. R^2^ represents the variance of the dependent variable explained by the model.

PLS-PM revealed that plant species had a significantly positive effect (*p* < 0.05) on soil physicochemical properties and exhibited a direct and significant positive correlation with the bacterial community in the topsoil (Figure 7). Conversely, there was a significant negative correlation between the soil physicochemical properties and the fungal community in the topsoil. Plant roots and soil metabolites had no significant direct effect on the microbial community in the topsoil. In the subsoil, plant roots directly and significantly positively affected soil physicochemical properties and fungal communities. Soil physicochemical properties had a significantly negative impact on the bacterial communities in the subsoil.

## 4 Discussion

### 4.1 Variation in soil physicochemical properties among the reforestation approaches

Previously, it was reported that reforestation could improve soil properties, but different stand forests have discrete impacts on the soil environment (He et al., 2021). Our results in the present study offer some novel information by comparing three artificial and natural reforestation approaches. The detection of varied nutrient (SOC, TN, DOC, and AP) concentrations in soils among the different reforestation approaches (Table 1) was consistent with that reported by He et al. (2021). The lower contents of SOC, TN, and AP in the pine forest than in the NR forest confirmed the findings of Zheng et al. (2005). However, the greater contents of SOC, TN, and AP in the topsoil of spruce and katsura plantations than those in NR (Figure 1) demonstrate that these artificially planted trees have improved soil fertility. Therefore, soil property improvement in the reforestation areas is a tree species-dependent procedure, which can affect the soil nutrient cycle and transformation process mainly through litter input and root activity (Hobbie, 2015; Angst et al., 2019).

The distinct characteristics of plant litter, including C/N and lignin/N ratios, directly affect the decomposition rate and soil inputs (Krishna and Mohan, 2017; Yang et al., 2022). Therefore, in the present study, the lower soil nutrients in pine forests may be related to the slower decomposition rates of pine needle litter than those of broad-leaved tree and shrub species (Li et al., 2009) since pine needle litter presents poorer quality, such as lower N concentration, higher C/N ratio, and greater lignin content. The lower SOC, TN, AP, and MBC concentrations in the subsoil than in the topsoil for all reforestation forests (Figure 1) are consistent with the conclusions of previous studies, such as He et al. (2021). Subsoil properties are also linked to root traits and diverse litterfalls of plants, as well as humus derived from plant residues (Zhu et al., 2020).

Differences in soil microbial community among the reforestation approaches Consistent with previous results (Wu et al., 2023; Zhang et al., 2023), the dominant bacterial phyla of the four reforestation approaches in the present study were Proteobacteria, Actinobacteria, and Acidobacteria, with variations in abundance. The significant differences in the diversity and composition of the bacterial and fungal communities revealed by LefSe (Figure 3) and PCoA (Figure 2) among the four reforestation approaches were consistent with the results of previous studies (Oria-de-Rueda et al., 2010; Yang et al., 2020). This might be due to the fact that different plant species result in different rhizosphere effects and rates of litter decomposition and nutrient release, which directly or indirectly enrich or deplete some specific microorganisms (Berg and Smalla, 2009; Vezzani et al., 2018; Xu et al., 2023). In the present study, the significant enrichment of Actinobacteria and Ascomycota in NR and katsura topsoils and of Proteobacteria (including Alphaproteobacteria) and Basidiomycota in spruce and pine topsoils (Figures 2A,B) might provide evidence for the effects of plant litter on soil microbial communities. In general, the litter of broad-leaved trees in the NR and katsura forests presented a lower C/N ratio and lignin content but greater cellulose content compared with that of the coniferous forests (spruce and pine). Members of Actinobacteria and Ascomycota are well-known degraders of cellulose. In addition, many members of Actinobacteria are plant growth-promoting bacteria that improve the availability of nutrients and minerals, synthesize plant growth regulators, and inhibit phytopathogens (Franco-Correa and Chavarro-Anzola, 2016). Corresponding to the high C/N ratio and high content of lignin (lignocellulose) in coniferous litter, Proteobacteria might be enriched in the topsoils of spruce and pine forests based on their nitrogen-fixing ability, which plays a crucial role in nitrogen recycling, which is beneficial for plant growth, yield, and the quality of fruits and seeds (Zhang H. et al., 2022). Members of fungal phylum Basidiomycota are well-known degraders of lignocellulose, which might be the main reason why they are enriched in spruce and pine topsoils. In subsoils, the microbial communities seemed not to be related to the litter characteristics but were more affected by the root systems in the four reforestation approaches since some similar compositions were detected between the forests of spruce and katsura and between the forests of NR and pine in both the bacterial and fungal communities. Further comparative studies on root exudates may explain the similarities in the soil microbial communities.

### 4.2 Soil metabolites and enriched metabolic pathways in different reforestation approaches

Metabolomics can reveal material cycling in soil and provide crucial insights into soil quality (Liu et al., 2021). The biological functions and co-network interactions of soil metabolites vary according to plant species and land use patterns (Mueller et al., 2020; Yao et al., 2023). However, there is insufficient information on soil metabolite fingerprints using different reforestation approaches. Our soil metabolite analysis results clearly showed that, in both soil layers, the artificial mono-plantations downregulated the expression of most DEMs compared to NR (Figure 5; Supplementary Figure S6). These differences might be related to the effects of tree species (biodiversity) on soil metabolites since four plant species were dominant in the NR forest, while the artificial forests were dominated by mono-planted species. Different plant species produce root exudates with distinct quantities and qualities (El Zahar Haichar et al., 2014; Weinhold et al., 2022), which directly form a part of the soil metabolites. In addition, different organic compounds exist in the litter produced by different plant species (Krishna and Mohan, 2017) and are transformed into diverse soil metabolites by distinct microbial degraders and microbial interactions (Figures 2–4). Higher plant diversity under natural restoration conditions may increase root-derived organic inputs, litter quality, and decomposition rates (Eisenhauer et al., 2017; Zheng et al., 2021).

The DEMs between natural reforestation and artificial plantation were fatty acyls, phenols, carboxylic acids, and derivatives (Figures 5G–L), indicating that artificial plantations were different from natural reforestation in the synthesis of complex lipids, the defense mechanism of the plant and soil phosphorus availability (Schefe et al., 2011; Mueller et al., 2020). The contents of related metabolites in the linoleic acid metabolism and valine, leucine, and isoleucine biosynthesis pathways were mainly different in artificial plantations-NR (Supplementary Figure S7). These differences may be attributed to the fact that artificial plantations have shaped the microbial community and regulated plant immunity, enhancing resistance to biotic and abiotic stresses (Feng et al., 2023; Liu Y. et al., 2023). Previously, it has been reported that the content of 13-S-hydroxyoctadecadienoic acid in roots increased in respond to the soil salinity (Li et al., 2023), while 13-L-hydroperoxylinoleic acid was involved in metabolism of linoleic acid that caused repelling of zoospores of the phytopathogen *Phytophthora sojae* in soybean rhizosphere (Zhang Z. et al., 2022). Therefore, the downregulation of prominent DEMs 13-L-hydroperoxylinoleic acid and 13-S-hydroxyoctadecadienoic acid found in the present study in artificial plantations compared with those in the NR forest might imply a reduction of the disease and stress resistance in forests.

### 4.3 Influence of soil metabolites and physicochemical properties on microbial communities

In the study area, forest soil is deficient in nitrogen and phosphorus under natural conditions (Wu et al., 2012; Liu et al., 2015). Our study showed that soil physicochemical properties and root traits play important roles in regulating soil microbial communities since soil physicochemical properties DON, AP, and MBN, and root character SRL were the most important factors significantly correlated with the structure of bacterial and fungal communities (Figure 6). Meanwhile, the responds of dominant bacterial and fungal taxa to the soil and root traits varied (Supplementary Figure S8). In general, more soil characters presented significant effects on soil bacterial and fungal communities than the root traits, and bacterial community might be more sensitive than the fungal communities to the variations in both the soil and root traits (Supplementary Figure S8). The significant positive correlation of the bacterial phyla Proteobacteria, Actinorhizobia, Firmicutes and Bacteroidetes with contents of various soil nutrients (C, N, P) might be related to the fact that heterotrophic bacteria are dominant in these phyla. In addition, the significant negative correlation of Proteobacteria, Acidobacteria, and Verrucomicrobia with pH value demonstrated that most member in these phyla detected in the tested forestry might be acidophilic, while the members of Gemmetimonadetes involved in this study might be alkaliphilic as evidenced by its significant correlation with pH value (Mucsi et al., 2024). Moreover, the significant positive correlation of Actinorhizobia and Bacteroidetes might imply their main association with fine roots (Acharya et al., 2023). The significantly positive correlation of fungal phylum Ascomycota with DON, AP, and MBN was consistent with its adaption to high-N soils (Wu et al., 2021) and high AP (Kuzikova and Medvedeva, 2023). While its significant positive correlation with RTD might imply their association with roots of the tested plants as mycorrhizal fungi and as endophytes (Kyslynska et al., 2023). In contrast, the negative correlations of Basidiomycota with RTD and of Mortierellomycota with MBC and MBN might reflect their competition relationships with roots and with other microbes (bacteria), respectively in the studied forests.

Our study also found that soil metabolites play an essential role in the regulation of soil microbial communities. As prominent metabolite responders, 13-L-hydroperoxylinoleic acid, 13-S-hydroxyoctadecadienoic acid, homovanillin, and 9,10-epoxyoctadecenoic acid were strongly correlated with microbial taxa in the network (Figure 6). In terms of ecological functions, 13-L-hydroperoxylinoleic acid and 13-S-hydroxyoctadecadienoic acid can also be classified as antistress metabolites that correlate with a significant number of microbial taxa (Figure 6E), which is consistent with the results of previous studies (Zhang Z. et al., 2022; Li et al., 2023).

PLS-PM results showed that vegetation types were the main factors that drove the variation in microbial community since the plants could have direct and indirect effects on the soil microbial community through root effects and by regulating physicochemical properties in the soil. The fine roots of trees are primarily distributed in the topsoil (0–20-cm soil layer) (52–71%), which also presents a greater exudation of live roots and decomposition of dead roots (Matamala et al., 2003; Yang et al., 2018). The root effect might significantly affect the soil metabolite spectrum, soil nutrients, and microenvironment, which in turn causes further differences in soil microbial communities among the tested reforestation approaches. Plants growing in environments with limited nutrients employ root exudates as signals for soil microbes involved in nutrient acquisition (Dakora and Phillips, 2002; Zhao et al., 2021). We speculate that the roots in the subsoil interact more with root-associated microorganisms, such as mycorrhizal fungi, to improve nutrient acquisition in different reforestation approaches. Soil metabolites play a crucial role in regulating interactions between microorganisms and plants (Wang et al., 2019). In general, the metabolism of rhizosphere soil is affected by plant roots more than the bulk soil (Raaijmakers et al., 2009), which may be an important reason for our finding that plant root traits had no significant effects on soil metabolites. Our results revealed that some metabolite responders were strongly correlated with microbial taxa, whereas soil metabolites had no significant effects on soil bacterial and fungal communities. It has been suggested that soil microorganisms are more sensitive to soil nutrients and the microenvironment during forest restoration.

## 5 Conclusion

Our study investigated soil physicochemical properties, metabolic profiles, microbial community composition, and diversity between artificial plantations and NR. The results revealed that artificial mono-plantation significantly changed the soil physicochemical properties, metabolites, and microbial communities compared to NR. Soil physicochemical properties (such as DON, AP, and MBN) were significantly correlated with dominant bacterial and fungal taxa and significantly affected bacterial and fungal communities in soil. In addition, plant root-mediated soil physicochemical properties were the primary factors to explain the variations in microbial communities among different reforestation approaches. In general, the ensemble of soil metabolites was not significantly affect the soil microbial community; however, several metabolites, such as 13-L-hydroperoxylinoleic acid, 13-S-hydroxyoctadecadienoic acid, homovanillin, and 9,10-epoxyoctadecenoic acid showed strong correlations with differentially abundant microbial taxa. Therefore, the connection between soil metabolomics and the microbial community is crucial for understanding the interactions among plant, soil physicochemical properties and soil microbes and for improving forecasts of forest ecological restoration using a reforestation approach. Further detailed exploration of the relationship between core metabolites and soil microorganisms is needed.

## Data Availability

The datasets presented in this study can be found in online repositories. The names of the repository/repositories and accession number(s) can be found at: https://nmdc.cn/resource/genomics/metagenome/detail/NMDC40055004, NMDC40055004-40055027, https://nmdc.cn/resource/genomics/metagenome/detail/NMDC40055028, NMDC40055028-40055051.

## References

[ref1] Acharya S. M. Yee M. O. Diamond S. Andeer P. F. Baig N. F. Aladesanmi O. T.. (2023). Fine scale sampling reveals early differentiation of rhizosphere microbiome from bulk soil in young Brachypodium plant roots. ISME Commun. 3:54. doi: 10.1038/s43705-023-00265-1, PMID: 37280433 PMC10244434

[ref2] Anderson M. J. (2006). Distance-based tests for homogeneity of multivariate dispersions. Biometrics 62, 245–253. doi: 10.1111/j.1541-0420.2005.00440.x, PMID: 16542252

[ref3] Angst G. Mueller K. E. Eissenstat D. M. Trumbore S. Freeman K. H. Hobbie S. E.. (2019). Soil organic carbon stability in forests: distinct effects of tree species identity and traits. Glob. Chang. Biol. 25, 1529–1546. doi: 10.1111/gcb.14548, PMID: 30554462

[ref4] Berg G. Smalla K. (2009). Plant species and soil type cooperatively shape the structure and function of microbial communities in the rhizosphere. FEMS Microbiol. Ecol. 68, 1–13. doi: 10.1111/j.1574-6941.2009.00654.x, PMID: 19243436

[ref5] Beugnon R. Bu W. Bruelheide H. Davrinche A. Du J. Haider S.. (2023). Abiotic and biotic drivers of tree trait effects on soil microbial biomass and soil carbon concentration. Ecol. Monogr. 93:e1563. doi: 10.1002/ecm.1563

[ref6] Bi B. Wang K. Zhang H. Wang Y. Fei H. Pan R.. (2021). Plants use rhizosphere metabolites to regulate soil microbial diversity. Land Degrad. Dev. 32, 5267–5280. doi: 10.1002/ldr.4107

[ref7] Brown S. P. Clark S. L. Ford E. Mirza N. Odeh A. Schlarbaum S. E.. (2023). Convergent shifts in soil fungal communities associated with Fagaceae reforestation in the southern Appalachian Mountains. Forest Ecol. Manag. 531:120805. doi: 10.1016/j.foreco.2023.120805

[ref8] Chamard J. Faticov M. Blanchet F. G. Chagnon P. L. Laforest-Lapointe I. (2024). Interplay of biotic and abiotic factors shapes tree seedling growth and root-associated microbial communities. Commun. Biol. 7:360. doi: 10.1038/s42003-024-06042-7, PMID: 38519711 PMC10960049

[ref9] Chen J. Shen W. Xu H. Li Y. Luo T. (2019). The composition of nitrogen-fixing microorganisms correlates with soil nitrogen content during reforestation: a comparison between legume and non-legume plantations. Front. Microbiol. 10:508. doi: 10.3389/fmicb.2019.00508, PMID: 30930882 PMC6427063

[ref10] Cheng H. Yuan M. Tang L. Shen Y. Yu Q. Li S. (2022). Integrated microbiology and metabolomics analysis reveal responses of soil microorganisms and metabolic functions to phosphorus fertilizer on semiarid farm. Sci. Total Environ. 817:152878. doi: 10.1016/j.scitotenv.2021.152878, PMID: 34998744

[ref11] Cui B. Liu X. Yang Q. Li J. Zhou X. Peng Y. (2017). Achieving partial denitrification through control of biofilm structure during biofilm growth in denitrifying biofilter. Bioresour. Technol. 238, 223–231. doi: 10.1016/j.biortech.2017.04.034, PMID: 28433912

[ref12] Dakora F. D. Phillips D. A. (2002). “Root exudates as mediators of mineral acquisition in low-nutrient environments” in Food security in nutrient-stressed environments: exploiting plants’ genetic capabilities (Berlin: Springer Science & Business Media), 201–213.

[ref13] Dhungana I. Kantar M. B. Nguyen N. H. (2023). Root exudate composition from different plant species influences the growth of rhizosphere bacteria. Rhizosphere 25:100645. doi: 10.1016/j.rhisph.2022.100645

[ref14] Eisenhauer N. Lanoue A. Strecker T. Scheu S. Steinauer K. Thakur M.. (2017). Root biomass and exudates link plant diversity with soil bacterial and fungal biomass. Sci. Rep. 7:44641. doi: 10.1038/srep44641, PMID: 28374800 PMC5379681

[ref15] El Zahar Haichar F. Santaella C. Heulin T. Achouak W. (2014). Root exudates mediated interactions belowground. Soil Biol. Biochem. 77, 69–80. doi: 10.1016/j.soilbio.2014.06.017

[ref16] Feng Z. Xie X. Wu P. Chen M. Qin Y. Zhou Y.. (2023). Phenylalanine-mediated changes in the soil bacterial community promote nitrogen cycling and plant growth. Microbiol. Res. 275:127447. doi: 10.1016/j.micres.2023.127447, PMID: 37441843

[ref17] FRA. (2010). Global forest resources assessment 2010: main report. Available online at: http://www.fao.org/docrep/013/i1757e/i1757e.pdf

[ref18] Franco-Correa M. Chavarro-Anzola V. (2016). “Actinobacteria as plant growth promoting rhizobacteria,” in Actinobacteria, Chapter 10. eds. D. Dhanasekaran and Y. Jiang.

[ref19] Hao C. Dungait J. A. Wei X. Ge T. Kuzyakov Y. Cui Z.. (2022). Maize root exudate composition alters rhizosphere bacterial community to control hotspots of hydrolase activity in response to nitrogen supply. Soil Biol. Biochem. 170:108717. doi: 10.1016/j.soilbio.2022.108717

[ref20] He Y. Han X. Wang X. Wang L. Liang T. (2021). Long-term ecological effects of two artificial forests on soil properties and quality in the eastern Qinghai-Tibet plateau. Sci. Total Environ. 796:148986. doi: 10.1016/j.scitotenv.2021.148986, PMID: 34274659

[ref21] Hobbie S. E. (2015). Plant species effects on nutrient cycling: revisiting litter feedbacks. Trends Ecol. Evol. 30, 357–363. doi: 10.1016/j.tree.2015.03.015, PMID: 25900044

[ref22] Hu B. Yang B. Pang X. Bao W. Tian G. (2016). Responses of soil phosphorus fractions to gap size in a reforested spruce forest. Geoderma 279, 61–69. doi: 10.1016/j.geoderma.2016.05.023

[ref23] Jiang T. T. Shao T.-Y. Ang W. G. Kinder J. M. Turner L. H. Pham G.. (2017). Commensal fungi recapitulate the protective benefits of intestinal bacteria. Cell Host Microbe 22:e804, 809–816.e4. doi: 10.1016/j.chom.2017.10.013, PMID: 29174402 PMC5730478

[ref24] Kaźmierczak M. Błońska E. Lasota J. (2024). Effect of litter decomposition and nutrient release from shrub litter on enzymatic activity and C/N/P stoichiometry of soils in a temperate pine forest. Acta Oecol. 124:104020. doi: 10.1016/j.actao.2024.104020

[ref25] Krishna M. Mohan M. (2017). Litter decomposition in forest ecosystems: a review. Energy Ecol. Environ. 2, 236–249. doi: 10.1007/s40974-017-0064-9

[ref26] Kuzikova I. L. Medvedeva N. G. (2023). Long-chain alkylphenol biodegradation potential of soil Ascomycota. Dokl. Biol. Sci. 511, 228–234. doi: 10.1134/S0012496623700515, PMID: 37833577 PMC10748767

[ref27] Kyslynska A. S. Nadkernychna O. V. Kopylov Y. P. Tsekhmister H. V. (2023). The relation between mutualistic mycorrhiza and endophytic plant-fungus associations and their effect on host plants. Agric. Sci. Practice 10, 54–75. doi: 10.15407/agrisp10.01.054

[ref28] Li W. Pan K.-W. Wu N. Wang J.-C. Han C.-M. Liang X.-L. (2009). Effects of mixing pine and broadleaved tree/shrub litter on decomposition and N dynamics in laboratory microcosms. Ecol. Res. 24, 761–769. doi: 10.1007/s11284-008-0546-5

[ref29] Li N. Shao T. Jia B. Yan X. Wang X. Tao C.. (2023). Amelioration of saline-alkali land by cultivating *Melia azedarach* and characterization of underlying mechanisms via metabolome analysis. Land Degrad. Dev. 34, 5556–5565. doi: 10.1002/ldr.4864

[ref30] Liu W. Liu Y. Wu S. Liu F. Wen Y. Wang L.. (2023). Dynamics of plant nutrient requirements and acquisition strategies after afforestation: a study on the loess plateau, China. For. Ecol. Manag. 544:121141. doi: 10.1016/j.foreco.2023.121141, PMID: 39959702

[ref31] Liu Y. Qin F. Li L. Dong X. Liu L. Yang L. (2024). The Long-term effects of barren land afforestation on plant productivity, soil fertility, and soil moisture in China: a meta-analysis. Plan. Theory 13:1614. doi: 10.3390/plants13121614, PMID: 38931046 PMC11207343

[ref32] Liu L. Wang T. Li S. Hao R. Li Q. (2021). Combined analysis of microbial community and microbial metabolites based on untargeted metabolomics during pig manure composting. Biodegradation 32, 217–228. doi: 10.1007/s10532-021-09935-0, PMID: 33710458

[ref33] Liu C. Wang Y. Pan K. Jin Y. Li W. Zhang L. (2015). Effects of phosphorus application on photosynthetic carbon and nitrogen metabolism, water use efficiency and growth of dwarf bamboo (*Fargesia rufa*) subjected to water deficit. Plant Physiol. Biochem. 96, 20–28. doi: 10.1016/j.plaphy.2015.07.018, PMID: 26218549

[ref34] Liu Y. Wilson A. J. Han J. Hui A. O’Sullivan L. Huan T.. (2023). Amino acid availability determines plant immune homeostasis in the rhizosphere microbiome. MBio 14, e03424–e03422. doi: 10.1128/mbio.03424-22, PMID: 36786577 PMC10127609

[ref35] Matamala R. Gonzalez-Meler M. A. Jastrow J. D. Norby R. J. Schlesinger W. H. (2003). Impacts of fine root turnover on forest NPP and soil C sequestration potential. Science 302, 1385–1387. doi: 10.1126/science.1089543, PMID: 14631037

[ref36] Morton J. T. Aksenov A. A. Nothias L. F. Foulds J. R. Quinn R. A. Badri M. H.. (2019). Learning representations of microbe–metabolite interactions. Nat. Methods 16, 1306–1314. doi: 10.1038/s41592-019-0616-3, PMID: 31686038 PMC6884698

[ref37] Mucsi M. Borsodi A. K. Megyes M. Szili-Kovács T. (2024). Response of the metabolic activity and taxonomic composition of bacterial communities to mosaically varying soil salinity and alkalinity. Sci. Rep. 14:7460. doi: 10.1038/s41598-024-57430-2, PMID: 38553497 PMC10980690

[ref38] Mueller L. O. Borstein S. R. Tague E. D. Dearth S. P. Castro H. F. Campagna S. R.. (2020). Populations of *Populus angustifolia* have evolved distinct metabolic profiles that influence their surrounding soil. Plant Soil 448, 399–411. doi: 10.1007/s11104-019-04405-2

[ref39] Olsen S. R. (1954). Estimation of available phosphorus in soils by extraction with sodium bicarbonate. Washington, DC: US Department of Agriculture.

[ref40] Oria-de-Rueda J. A. Hernández-Rodríguez M. Martín-Pinto P. Pando V. Olaizola J. (2010). Could artificial reforestations provide as much production and diversity of fungal species as natural forest stands in marginal Mediterranean areas? For. Ecol. Manag. 260, 171–180. doi: 10.1016/j.foreco.2010.04.009

[ref41] Philippot L. Chenu C. Kappler A. Rillig M. C. Fierer N. (2023). The interplay between microbial communities and soil properties. Nat. Rev. Microbiol. 22, 226–239. doi: 10.1038/s41579-023-00980-5, PMID: 37863969

[ref9001] Raaijmakers J. M. Paulitz T. C. Steinberg C. Alabouvette C. Moënne-Loccoz Y. (2009). The rhizosphere: a playground and battlefield for soilborne pathogens and beneficial microorganisms. Plant Soil 321, 341–361. doi: 10.1007/s11104-008-9568-6

[ref42] Sakti A. D. Komara H. Y. Sumarga E. Choiruddin A. Hendrawan V. S. A. Hati T.. (2024). Optimizing afforestation and reforestation strategies to enhance ecosystem services in critically degraded regions. Trees Forests People 18:100700. doi: 10.1016/j.tfp.2024.100700

[ref43] Sanchez G. (2013). PLS path modeling with R, vol. 383. Berkeley: Trowchez Editions, 551.

[ref44] Schefe C. Kappen P. Pigram P. J. (2011). Carboxylic acids affect sorption and micro-scale distribution of phosphorus in an acidic soil. Soil Sci. Soc. Am. J. 75, 35–44. doi: 10.2136/sssaj2010.0068

[ref45] Segata N. Izard J. Waldron L. Gevers D. Miropolsky L. Garrett W. S.. (2011). Metagenomic biomarker discovery and explanation. Genome Biol. 12, 1–18. doi: 10.1186/gb-2011-12-s1-p47, PMID: 21702898 PMC3218848

[ref46] Shi X. Zhao Y. Xu M. Ma L. Adams J. M. Shi Y. (2024). Insights into plant–microbe interactions in the rhizosphere to promote sustainable agriculture in the new crops era. New Crops 1:100004. doi: 10.1016/j.ncrops.2023.11.002

[ref47] Song Y. Li X. Yao S. Yang X. Jiang X. (2020). Correlations between soil metabolomics and bacterial community structures in the pepper rhizosphere under plastic greenhouse cultivation. Sci. Total Environ. 728:138439. doi: 10.1016/j.scitotenv.2020.138439, PMID: 32361108

[ref48] Spitzer C. M. Lindahl B. Wardle D. A. Sundqvist M. K. Kardol P. (2021). Root trait-microbial relationships across tundra plant species. New Phytol. 229, 1508–1520. doi: 10.1111/nph.16982, PMID: 33007155 PMC7821200

[ref49] Vance E. D. Brookes P. C. Jenkinson D. S. (1987). An extraction method for measuring soil microbial biomass C. Soil Biol. Biochem. 19, 703–707. doi: 10.1016/0038-0717(87)90052-6

[ref50] Vasilev N. Boccard J. Lang G. Grömping U. Fischer R. Goepfert S.. (2016). Structured plant metabolomics for the simultaneous exploration of multiple factors. Sci. Rep. 6:37390. doi: 10.1038/srep37390, PMID: 27853298 PMC5112604

[ref51] Veldkamp E. Schmidt M. Powers J. S. Corre M. D. (2020). Deforestation and reforestation impacts on soils in the tropics. Nature Rev. Earth Environ. 1, 590–605. doi: 10.1038/s43017-020-0091-5

[ref52] Vezzani F. M. Anderson C. Meenken E. Gillespie R. Peterson M. Beare M. H. (2018). The importance of plants to development and maintenance of soil structure, microbial communities and ecosystem functions. Soil Tillage Res. 175, 139–149. doi: 10.1016/j.still.2017.09.002

[ref53] Wang Y. Ren W. Li Y. Xu Y. Teng Y. Christie P.. (2019). Nontargeted metabolomic analysis to unravel the impact of di (2-ethylhexyl) phthalate stress on root exudates of alfalfa (*Medicago sativa*). Sci. Total Environ. 646, 212–219. doi: 10.1016/j.scitotenv.2018.07.247, PMID: 30053665

[ref54] Wang J. Wu Y. Zhou J. Bing H. Sun H. (2016). Carbon demand drives microbial mineralization of organic phosphorus during the early stage of soil development. Biol. Fertil. Soils 52, 825–839. doi: 10.1007/s00374-016-1123-7

[ref56] Weinhold A. Döll S. Liu M. Schedl A. Pöschl Y. Xu X.. (2022). Tree species richness differentially affects the chemical composition of leaves, roots and root exudates in four subtropical tree species. J. Ecol. 110, 97–116. doi: 10.1111/1365-2745.13777

[ref57] Wille L. Messmer M. M. Studer B. Hohmann P. (2019). Insights to plant–microbe interactions provide opportunities to improve resistance breeding against root diseases in grain legumes. Plant Cell Environ. 42, 20–40. doi: 10.1111/pce.13214, PMID: 29645277

[ref58] WRB I.-W. (2006). World reference base for soil resources. World Soil Resources Rep. 103, 1–128.

[ref59] Wu F. Bao W. Zhou Z. Li F. (2012). Appropriate nitrogen supply could improve soil microbial and chemical characteristics with *Sophora davidii* seedlings cultivated in water stress conditions. Acta Agriculturae Scandinavica Section B-Soil Plant Sci 62, 49–58. doi: 10.1080/09064710.2011.568515, PMID: 39935898

[ref60] Wu F. Wang Y. Sun H. Zhou J. Li R. (2023). Reforestation regulated soil bacterial community structure along vertical profiles in the loess plateau. Front. Microbiol. 14:1324052. doi: 10.3389/fmicb.2023.1324052, PMID: 38088965 PMC10713748

[ref61] Wu X. Zhang T. Zhao J. Wang L. Yang D. Li G.. (2021). Variation of soil bacterial and fungal communities from fluvo-aquic soil under chemical fertilizer reduction combined with organic materials in North China plain. J. Soil Sci. Plant Nutr. 21, 349–363. doi: 10.1007/s42729-020-00365-0

[ref62] Xu Z. Hu Z. Jiao S. Bell S. M. Xu Q. Ma L.. (2023). Depth-dependent effects of tree species identity on soil microbial community characteristics and multifunctionality. Sci. Total Environ. 878:162972. doi: 10.1016/j.scitotenv.2023.162972, PMID: 36958562

[ref63] Yang B. Qi K. Bhusal D. R. Huang J. Chen W. Wu Q.. (2020). Soil microbial community and enzymatic activity in soil particle-size fractions of spruce plantation and secondary birch forest. Eur. J. Soil Biol. 99:103196. doi: 10.1016/j.ejsobi.2020.103196

[ref64] Yang K. Zhu J. Xu S. Zheng X. (2018). Conversion from temperate secondary forests into plantations (*Larix spp.*): impact on belowground carbon and nutrient pools in northeastern China. Land Degrad. Dev. 29, 4129–4139. doi: 10.1002/ldr.3169

[ref65] Yang K. Zhu J. Zhang W. Zhang Q. Lu D. Zhang Y.. (2022). Litter decomposition and nutrient release from monospecific and mixed litters: comparisons of litter quality, fauna and decomposition site effects. J. Ecol. 110, 1673–1686. doi: 10.1111/1365-2745.13902

[ref66] Yao S. Bian Y. Jiang X. Song Y. (2023). Characterization of dissolved organic matter distribution in forestland and farmland of mollisol based on untargeted metabolomics. Soil Ecol. Lett. 5:230179. doi: 10.1007/s42832-023-0179-1

[ref67] Zelena E. Dunn W. B. Broadhurst D. Francis-McIntyre S. Carroll K. M. Begley P.. (2009). Development of a robust and repeatable UPLC− MS method for the long-term metabolomic study of human serum. Anal. Chem. 81, 1357–1364. doi: 10.1021/ac8019366, PMID: 19170513

[ref68] Zhang Z. Bi X. Du X. Liu H. An T. Zhao Y.. (2022). Comparative metabolomics reveal the participation of soybean unique rhizosphere metabolites in susceptibility and resistance of host soybean to *Phytophthora sojae*. Plant Soil 480, 185–199. doi: 10.1007/s11104-022-05571-6

[ref69] Zhang N. N. Chen X. X. Liang J. Zhao C. Xiang J. Luo L.. (2023). Rhizocompartmental microbiomes of arrow bamboo (*Fargesia nitida*) and their relation to soil properties in subalpine coniferous forests. PeerJ 11:e16488. doi: 10.7717/peerj.16488, PMID: 38047031 PMC10693234

[ref70] Zhang H. Li Y. Xu Y. John R. (2024). The recovery of soil N-cycling and P-cycling following reforestation in a degraded tropical limestone mine. J. Clean. Prod. 448:141580. doi: 10.1016/j.jclepro.2024.141580

[ref71] Zhang H. Ullah F. Ahmad R. Shah S. U. A. Khan A. Adnan M. (2022). Response of soil proteobacteria to biochar amendment in sustainable agriculture-a mini review. J. Soil Plant Environ. 1, 16–30. doi: 10.56946/jspae.v1i2.56

[ref72] Zhao M. Zhao J. Yuan J. Hale L. Wen T. Huang Q.. (2021). Root exudates drive soil-microbe-nutrient feedbacks in response to plant growth. Plant Cell Environ. 44, 613–628. doi: 10.1111/pce.13928, PMID: 33103781

[ref73] Zheng H. Ouyang Z. Wang X. Miao H. Zhao T. Peng T. (2005). How different reforestation approaches affect red soil properties in southern China. Land Degrad. Dev. 16, 387–396. doi: 10.1002/ldr.650

[ref74] Zheng H. Yang T. Bao Y. He P. Yang K. Mei X.. (2021). Network analysis and subsequent culturing reveal keystone taxa involved in microbial litter decomposition dynamics. Soil Biol. Biochem. 157:108230. doi: 10.1016/j.soilbio.2021.108230

[ref75] Zhu L. Wang J. Weng Y. Chen X. Wu L. (2020). Soil characteristics of *Eucalyptus urophylla*× *Eucalyptus grandis* plantations under different management measures for harvest residues with soil depth gradient across time. Ecol. Indic. 117:106530. doi: 10.1016/j.ecolind.2020.106530

