# Supplementary material for: Differentiation of soil metabolic function and microbial communities between plantations and natural reforestation
Source: Front Microbiol. 2025 Feb 28;16:1544641. doi: 10.3389/fmicb.2025.1544641 (PMC11906678; doi:10.3389/fmicb.2025.1544641)
Supplement: Supplementary file 1 [file Table_1.docx]

**Supplementary Text 1**

The PCR amplification program included an initial denaturation at 95 °C for 2 min, followed by 25 cycles of 94 °C for 40 s, 50 °C for 60 s, and 72 °C for 60 s, and a final extension at 72 °C for 10 min. The PCR components contained 5 μl of 5× buffer, 2 μL of 2.5 mM dNTPs, 0.25 μl of 5U/μl Fast pfu DNA Polymerase, 1 μL of each primer (5 μM), 1 μl of DNA template, and 14.75 μl of ddH2O. Amplificons in triplicate purified with Vazyme VAHTSTM DNA Clean Beads (Vazyme, Nanjing, China) and quantified by the Quant-iT PicoGreen dsDNA Assay Kit (Invitrogen, Carlsbad, CA, USA). Purified amplificons were pooled in equimolar and paired-end sequenced (2 × 250) on an Illumina MiSeq platform at Shanghai Personal Biotechnology Co., Ltd (Shanghai, China).

Raw fastq files were demultiplexed and quality-filtered using QIIME2 2019.4 (Bolyen *et al.*, 2019) (http://docs.qiime2.org/2019.4/tutorials/) with slight modification. Briefly, after paired-end merging, sequences were quality filtered and chimera removed was using the DADA2 plugin (Callahan *et al.*, 2016). Non-singleton amplicon sequence variants were aligned using mafft (Katoh *et al.*, 2002) and used to build a phylogenetic tree with fasttree2 (Price *et al.*, 2009). Alpha-diversity metrics (Shannon and Chao1) and Good’s coverage were estimated. The taxonomy affiliation was assigned against the bacterial SILVA Release 132 and fungal UNITE Release 8.0 database (Kõljalg *et al.*, 2013).


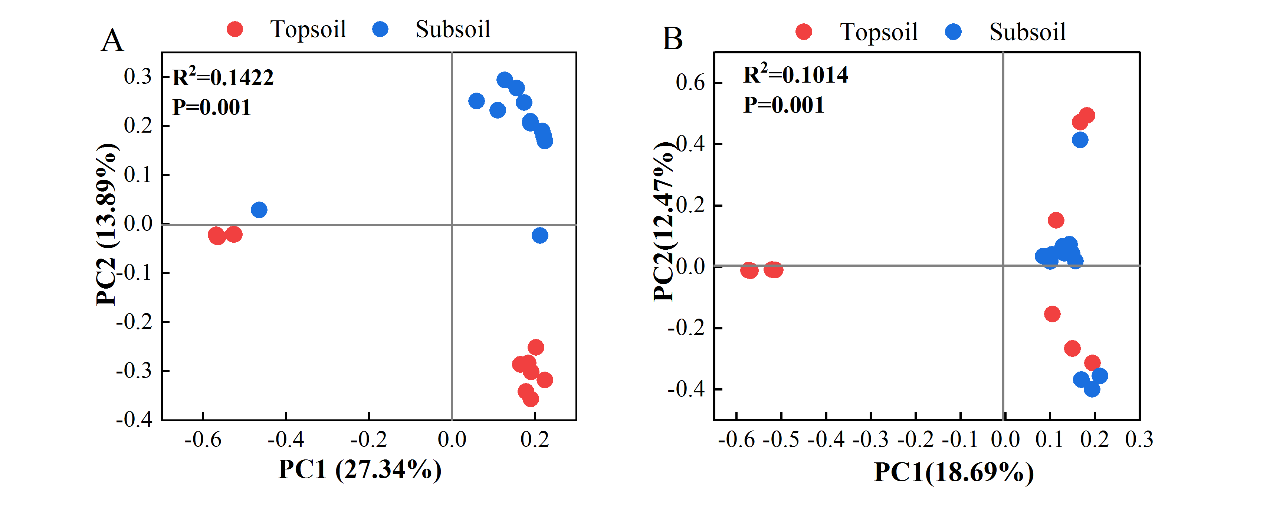


**Fig. S1**. Principal coordinate analysis (PCoA) illustrating changes in bacterial (A) and fungal (B) community structure in topsoil and subsoil.


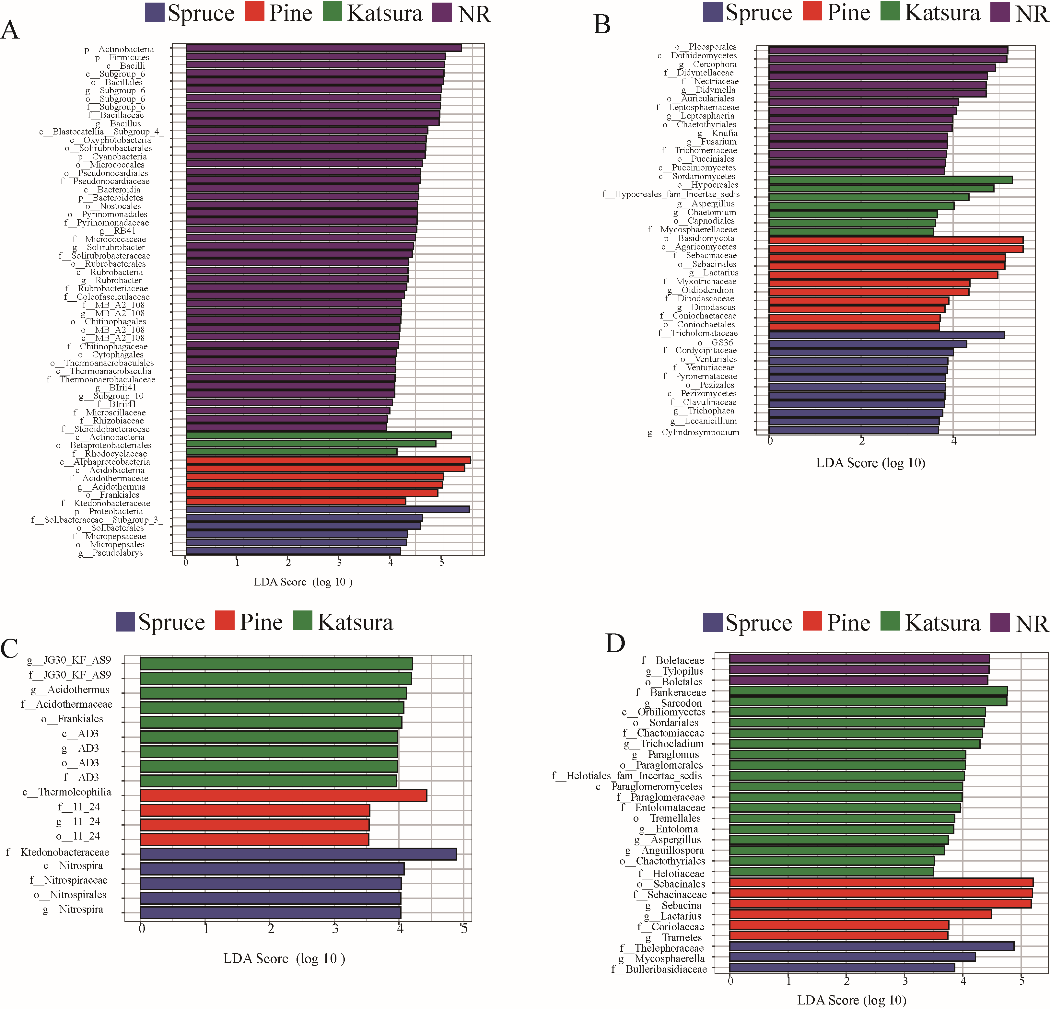


**Fig. S2**. Liner discriminant analysis, in conjunction with effect size measurements, identifies the differentially abundant taxa between different reforestation approaches of bacterial (**A, C**) and fungal taxa in topsoil and subsoil. Lineages with LDA values greater than 3.5 are shown.


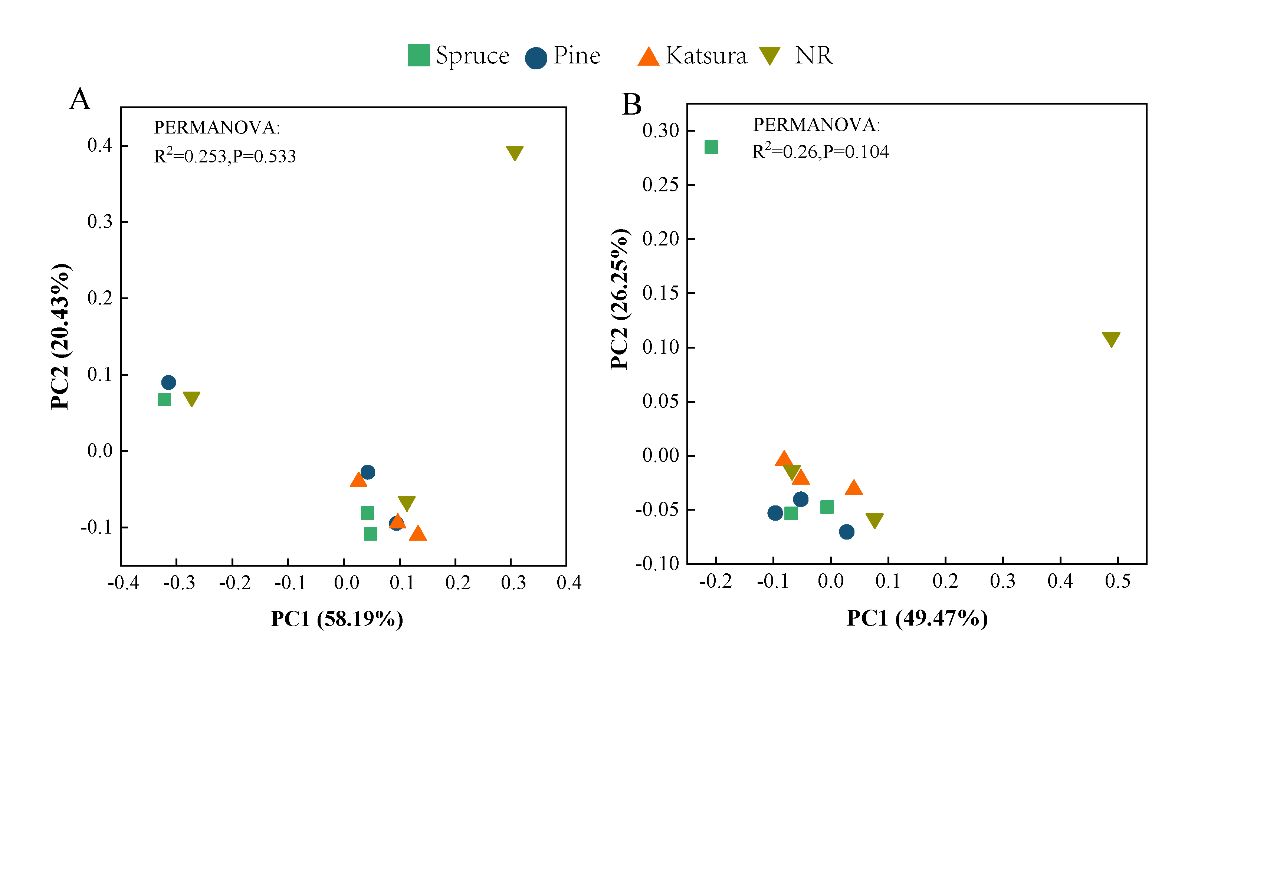


**
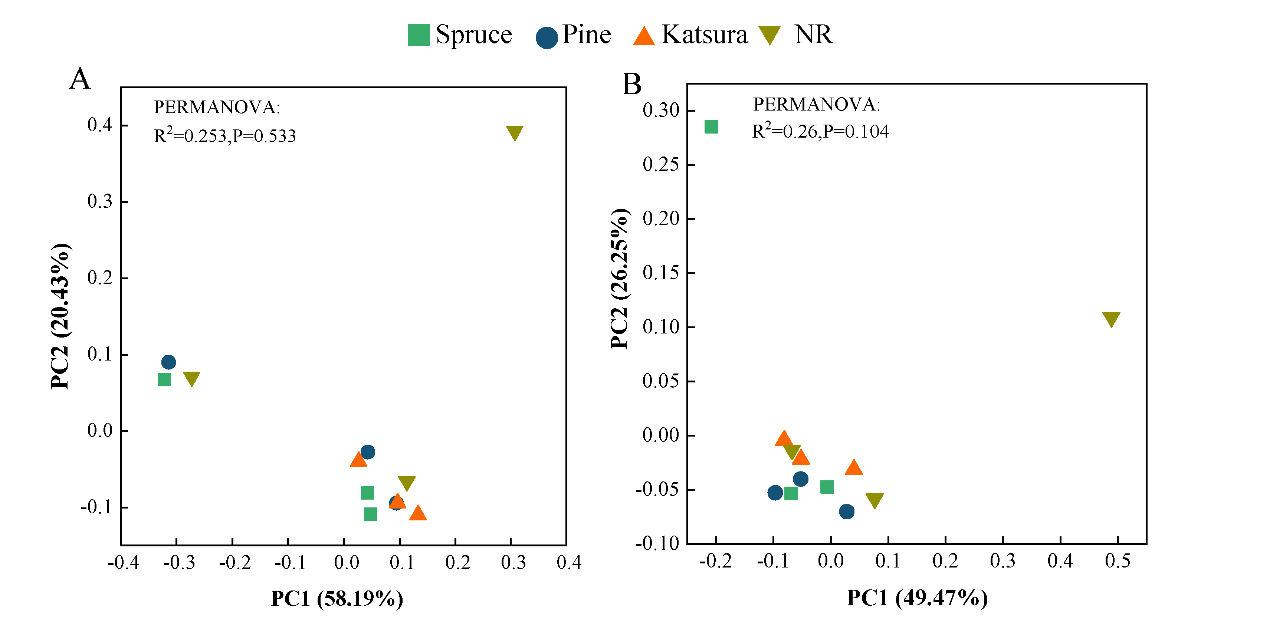
Fig. S3**. Principal coordinate analysis (PCoA) illustrating changes in metabolites profile in topsoil (A) and subsoil (B).


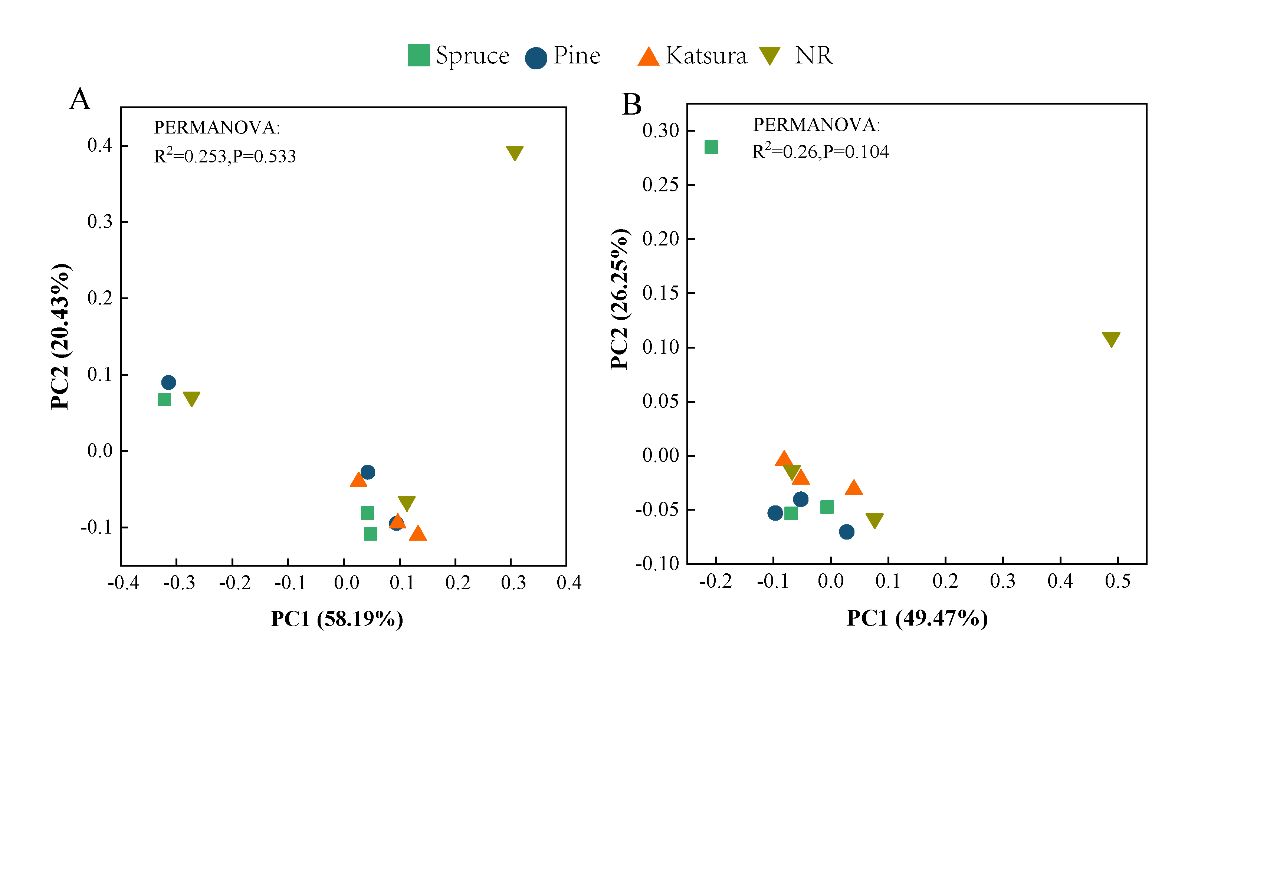


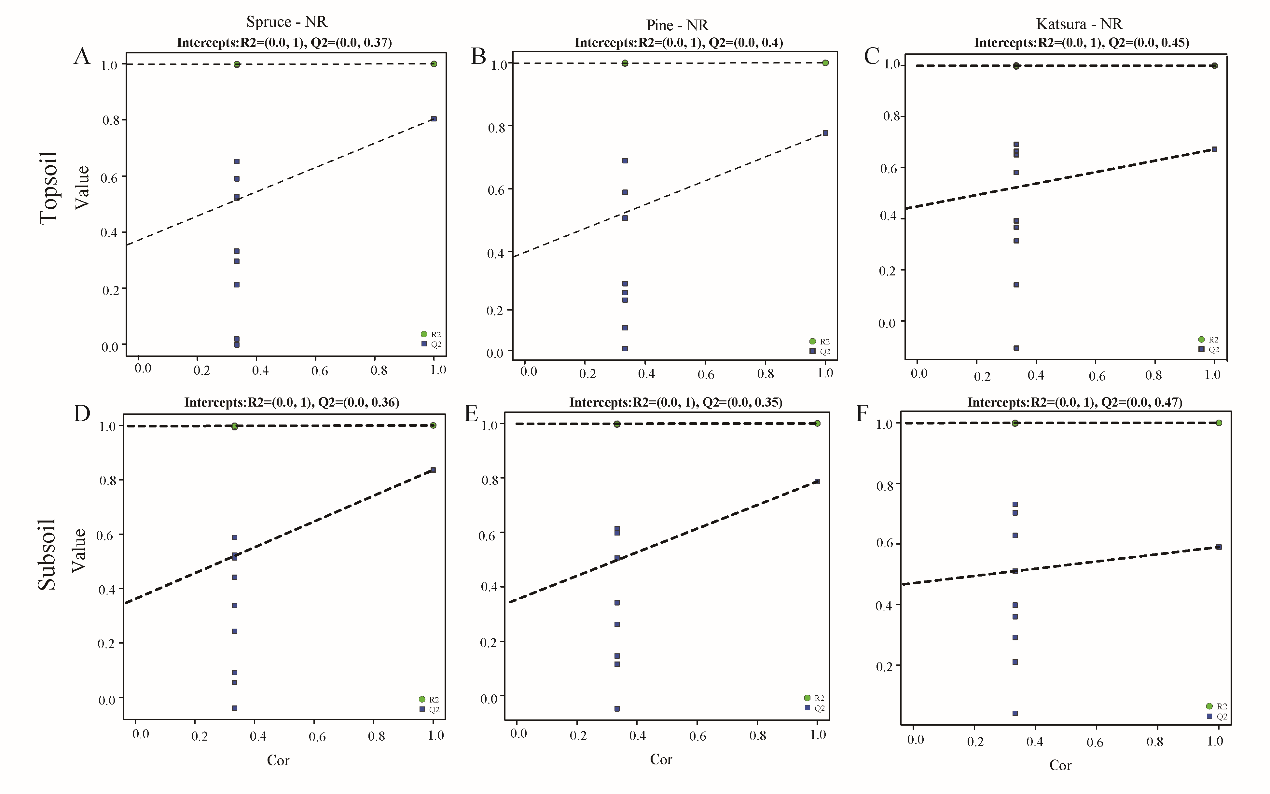


**Fig. S4**. Cross-validation model of PLS-DA between artificial plantations and NR in topsoil (A, B, C) and subsoil (D, E, F).


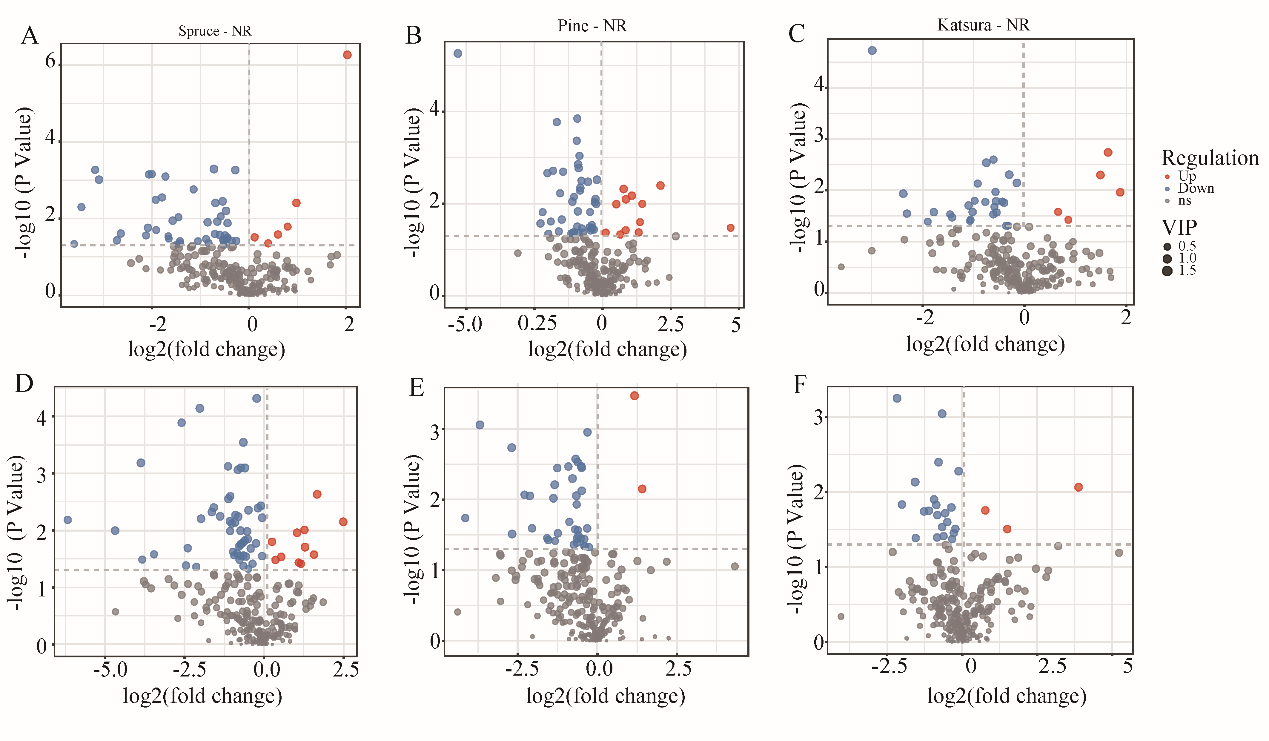


**Fig. S5**. The expression volcano map of differential metabolites up and down regulate. Blue dots represent down-regulated metabolites, red dots represent up-regulated metabolites, and gray dots represent no-differential metabolites.


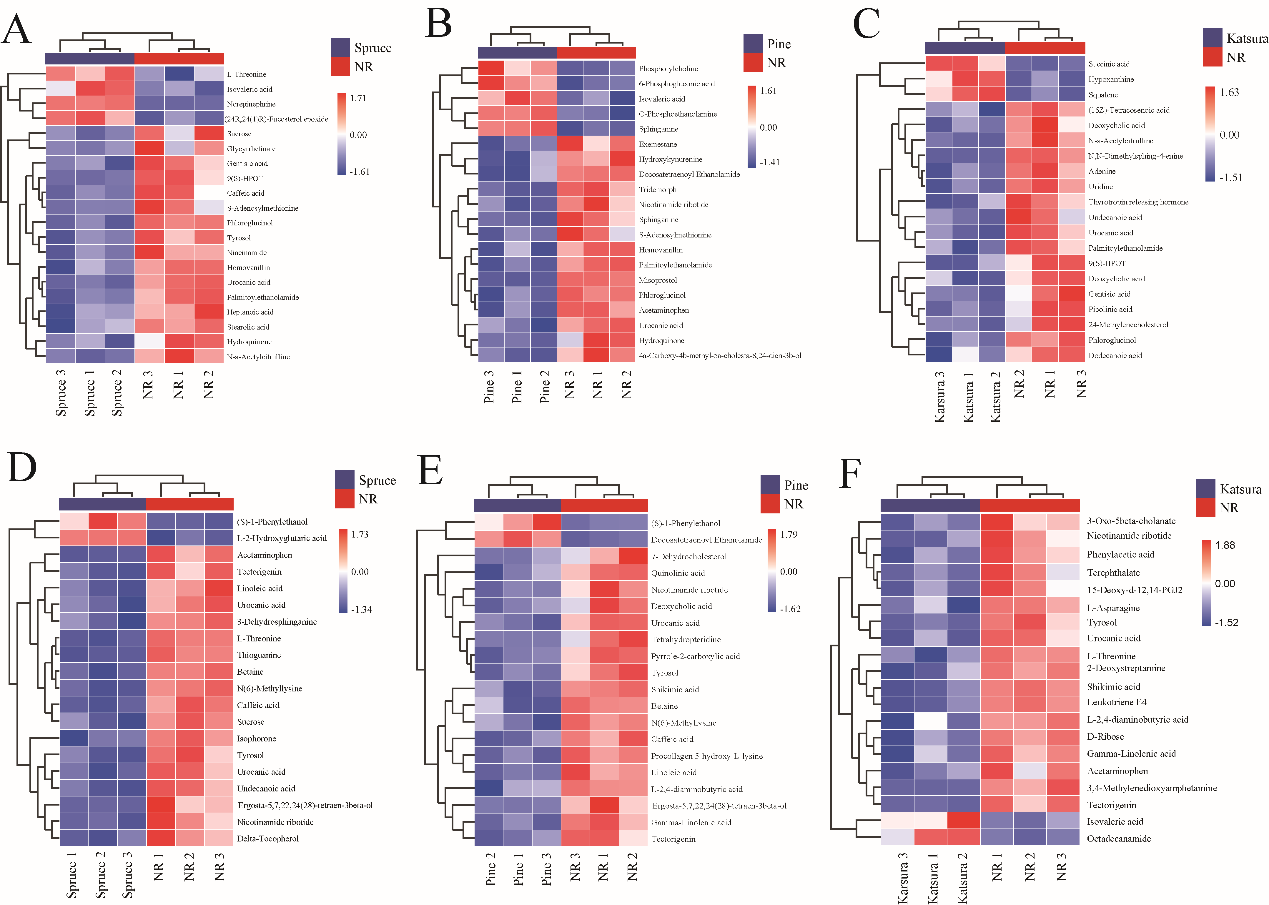


**Fig. S6**. Heat map analysis of the top 20 differential metabolites between artificial plantations and NR in topsoil (A, B, C) and subsoil (D, E, F).

.


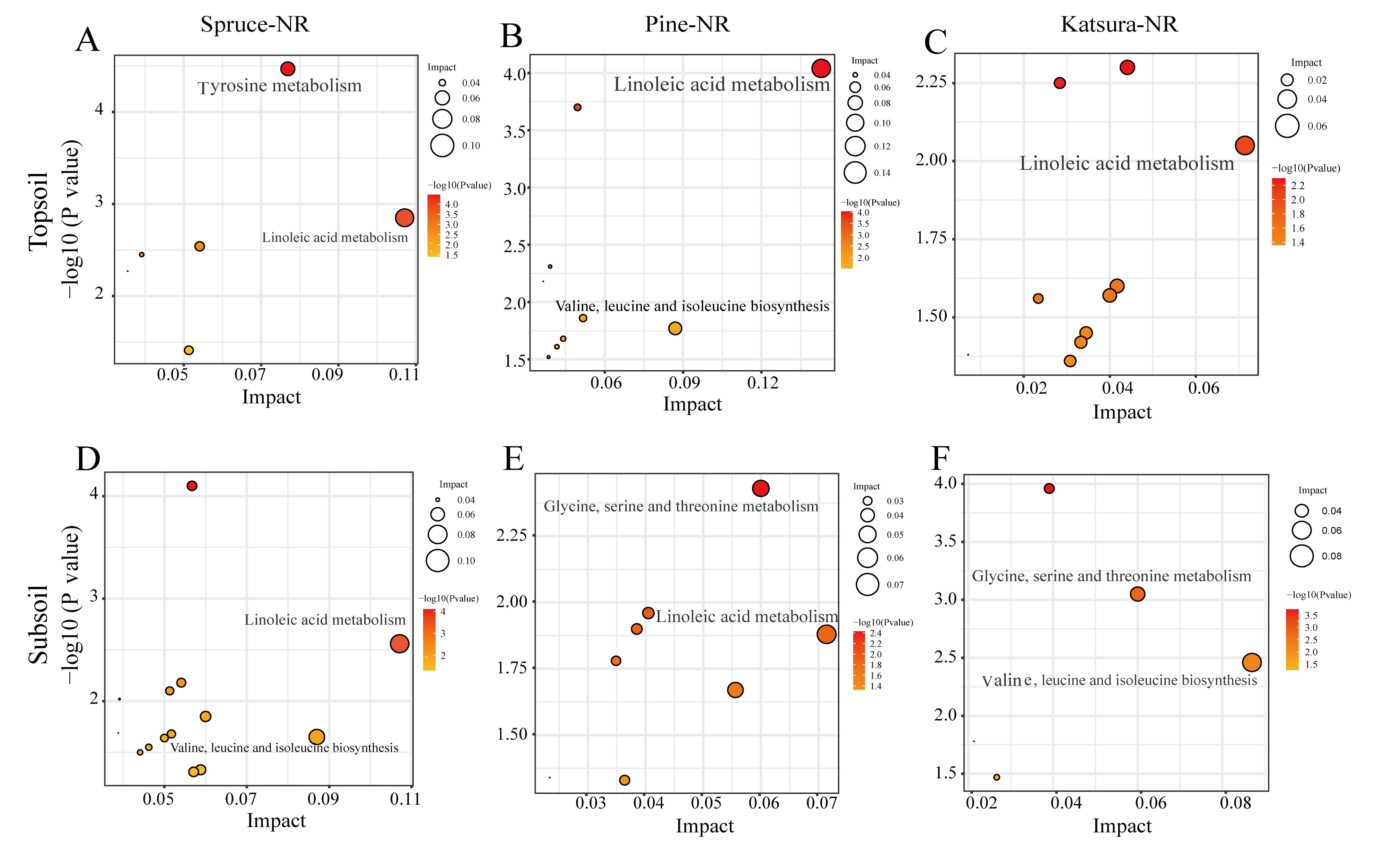


**Fig. S7**. Bubble diagram demonstrated the metabolic pathways that were significantly different between artificial plantations and NR in topsoil (A, B, C) and subsoil (D, E, F).


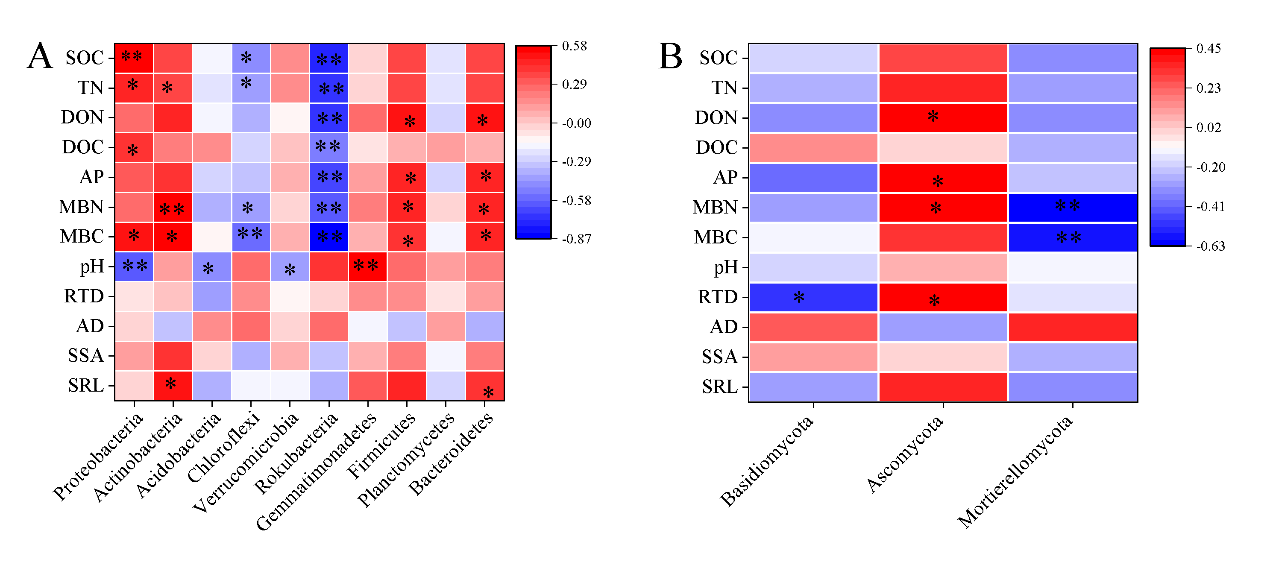


**Fig. S8**. Correlation of the dominant bacterial and fungal phyla, in terms of relative abundance, with soil physicochemical property and root traits. Red and blue indicate positive and negative correlation, respectively. * and ** represent significance (P < 0.05) and high significance (P < 0.01).

**Table S1**. Network topological features of the microbial networks.

| Topological properties | Bacteria | | | | Fungi | | | |
| --- | --- | --- | --- | --- | --- | --- | --- | --- |
|  | spruce | pine | katsura | NR | spruce | pine | katsura | NR |
| Nodes | 85 | 73 | 127 | 137 | 22 | 13 | 41 | 55 |
| Edge | 163 | 98 | 325 | 1228 | 18 | 10 | 46 | 249 |
| APL | 1.252 | 1.577 | 1.298 | 3.326 | 1.1 | 1.167 | 1.895 | 2.558 |
| ACC | 0.993 | 0.930 | 0.926 | 0.532 | 0.917 | 0.889 | 0.737 | 0.559 |
| Network density | 0.046 | 0.037 | 0.041 | 0.132 | 0.078 | 0.128 | 0.056 | 0.168 |
| Degree | 3.835 | 2.685 | 5.118 | 17.927 | 1.636 | 1.538 | 2.244 | 9.055 |
| Modularity | 2.737 | 2.189 | 5.905 | 5.566 | 1.084 | 0.72 | 1.292 | 0.758 |

APL: average path length; ACC: average clustering coefficient

**Table S2**. Differential metabolites of major metabolic pathways

| group | Metabolites ID | Metabolites | Super Class | |
| --- | --- | --- | --- | --- |
| Spruce-NR | Topsoil | | |  |
|  | M121T813 | Tyrosol | Phenols / down-regulated |  |
|  | M110T35_2 | Hydroquinone | Phenols/ down-regulated |  |
|  | M155T603 | Gentisic acid | Benzene and substituted derivatives / down-regulated |  |
|  | M152T290 | 3,4-Dihydroxyphenylacetaldehyde | Benzene and substituted derivatives / down-regulated |  |
|  | M167T938 | Homovanillin | Phenols / down-regulated |  |
|  | M170T654 | Norepinephrine | Phenols /up-regulated |  |
|  | M280T969 | Bovinic acid | Fatty Acyls / down-regulated |  |
|  | M295T975 | 13S-hydroxyoctadecadienoic acid | Fatty Acyls / down-regulated |  |
|  | M293T856 | 13-L-Hydroperoxylinoleic acid | Fatty Acyls / down-regulated |  |
|  | Subsoil | | |  |
|  | M278T451 | gamma-Linolenic acid | Fatty Acyls / down-regulated |  |
|  | M280T960 | Linoleic acid | Fatty Acyls / down-regulated |  |
|  | M119T139 | L-Threonine | Carboxylic acids and derivatives / down-regulated |  |
|  | M130T796 | L-Isoleucine | Carboxylic acids and derivatives /up-regulated |  |
|  | M295T835 | 9,10-Epoxyoctadecenoic acid | Fatty Acyls / down-regulated |  |
| Pine-NR | Topsoil | | |  |
|  | M295T835 | 9,10-Epoxyoctadecenoic acid | Fatty Acyls / down-regulated |  |
|  | M119T139 | L-Threonine | Carboxylic acids and derivatives /up-regulated |  |
|  | M116T791 | L-Valine | Carboxylic acids and derivatives / down-regulated |  |
|  | M313T800 | 9,10-DHOME | Fatty Acyls / down-regulated |  |
|  | M295T975 | 13S-hydroxyoctadecadienoic acid | Fatty Acyls / down-regulated |  |
|  | M293T856 | 13-L-Hydroperoxylinoleic acid | Fatty Acyls / down-regulated |  |
|  | Subsoil | | |  |
|  | M118T89 | Betaine | Carboxylic acids and derivatives / down-regulated |  |
|  | M278T451 | gamma-Linolenic acid | Fatty Acyls / down-regulated |  |
|  | M280T960 | Linoleic acid | Fatty Acyls / down-regulated |  |
|  | M102T70_3 | 2-Ketobutyric acid | Keto acid and derivatives / down-regulated |  |
|  | M118T782 | L-2,4-diaminobutyric acid | Carboxylic acids and derivatives / down-regulated |  |
| Katsura-NR | Topsoil | | |  |
|  | M295T835 | 9,10-Epoxyoctadecenoic acid | Fatty Acyls / down-regulated |  |
|  | M295T975 | 13S-hydroxyoctadecadienoic acid | Fatty Acyls / down-regulated |  |
|  | Subsoil | | |  |
|  | M119T139 | L-Threonine | Carboxylic acids and derivatives / down-regulated |  |
|  | M102T70_3 | 2-Ketobutyric acid | Keto acid and derivatives / down-regulated |  |
|  | M118T782 | L-2,4-diaminobutyr*ic aci*d | Carboxylic acids and derivatives / down-regulated |  |

Bolyen, E., Rideout, J.R., Dillon, M.R., Bokulich, N.A., Abnet, C.C., Al-Ghalith, G.A., Alexander, H., Alm, E.J., Arumugam, M., Asnicar, F.J.N.b., 2019. Reproducible, interactive, scalable and extensible microbiome data science using QIIME 2. 37, 852-857.

Callahan, B.J., McMurdie, P.J., Rosen, M.J., Han, A.W., Johnson, A.J.A., Holmes, S.P.J.N.m., 2016. DADA2: High-resolution sample inference from Illumina amplicon data. 13, 581-583.

Katoh, K., Misawa, K., Kuma, K.i., Miyata, T.J.N.a.r., 2002. MAFFT: a novel method for rapid multiple sequence alignment based on fast Fourier transform. 30, 3059-3066.

Kõljalg, U., Nilsson, R.H., Abarenkov, K., Tedersoo, L., Taylor, A.F., Bahram, M., Bates, S.T., Bruns, T.D., Bengtsson‐Palme, J., Callaghan, T.M., 2013. Towards a unified paradigm for sequence‐based identification of fungi. Wiley Online Library.

Price, M.N., Dehal, P.S., Arkin, A.P.J.M.b., evolution, 2009. FastTree: computing large minimum evolution trees with profiles instead of a distance matrix. 26, 1641-1650.
